# Investigating the Therapeutic Potential of Plants and Plant-Based Medicines: Relevance to Antioxidant and Neuroprotective Effects

**DOI:** 10.3390/nu15183912

**Published:** 2023-09-08

**Authors:** Naomi May, Julianna Lys de Sousa Alves Neri, Helen Clunas, Jiahua Shi, Ella Parkes, Anjila Dongol, Zhizhen Wang, Carlos Jimenez Naranjo, Yinghua Yu, Xu-Feng Huang, Karen Charlton, Katrina Weston-Green

**Affiliations:** 1 Molecular Horizons and School of Medical, Indigenous and Health Sciences, Faculty of Science, Medicine and Health, University of Wollongong, Wollongong, NSW 2522, Australia; 2 Australian Centre for Cannabinoid Clinical and Research Excellence, New Lambton Heights, NSW 2305, Australia; 3 Jiangsu Key Laboratory of Immunity and Metabolism, Department of Pathogen Biology and Immunology, Xuzhou Medical University, Xuzhou 221004, China

**Keywords:** oxidative stress, neuroprotection, cognition, novel therapeutics, psychiatric disorders, dementia, plant-based medicine, phenolic, flavonoid, anthocyanin

## Abstract

Oxidative stress is a common characteristic of psychiatric, neurological, and neurodegenerative disorders. Therefore, compounds that are neuroprotective and reduce oxidative stress may be of interest as novel therapeutics. Phenolic, flavonoid and anthocyanin content, ORAC and DPPH free radical scavenging, and Cu^2+^ and Fe^2+^ chelating capacities were examined in variations (fresh/capsule) of Queen Garnet plum (QGP, *Prunus salicina*), black pepper (*Piper nigrum*) clove (*Syzygium aromaticum*), elderberry (*Sambucus nigra*), lemon balm (*Melissa officinalis*) and sage (*Salvia officinalis*), plus two blends (*Astralagus membranaceus*—lemon balm-rich, WC and R8). The ability of samples to prevent and treat H_2_O_2_-induced oxidative stress in SH-SY5Y cells was investigated. Pre-treatment with WC, elderberry, QGP, and clove prevented the oxidative stress-induced reduction in cell viability, demonstrating a neuroprotective effect. Elderberry increased cell viability following oxidative stress induction, demonstrating treatment effects. Clove had the highest phenolic and flavonoid content, DPPH, and Cu^2+^ chelating capacities, whereas QGP and elderberry were highest in anthocyanins. Black pepper had the highest ORAC and Fe^2+^ chelating capacity. These findings demonstrate that plant extracts can prevent and treat oxidative stress-induced apoptosis of neuron-like cells in vitro. Further research into phytochemicals as novel therapeutics for oxidative stress in the brain is needed.

## 1. Introduction

Oxidative stress results from excessive levels of reactive oxygen species (ROS) and reactive nitrogen species (RNS) that can damage cell structures, particularly the mitochondria, leading to the accumulation of cytotoxic mediators, cell death, and inflammation [1]. Oxidative damage can also be induced by transition metals, such as copper and iron, that reduce oxygen, leading to the generation of ROS and subsequent oxidation of proteins, lipids, and nucleic acids [2]. The brain is particularly susceptible to ROS actions due to its high lipid content, high metabolic activity, non-replicative nature, and weak antioxidant capacity [1]. Indeed, growing research suggests that inflammation and oxidative stress are associated with psychiatric and neurodegenerative disorders [3,4]. For example, significantly elevated levels of peripheral markers of oxidative stress have been reported in people with Mild Cognitive Impairment (MCI) compared to healthy controls [5], and oxidative stress has been identified as a factor that predicts accelerated cognitive decline with aging [6,7]. Furthermore, a meta-analysis of eighty-two studies showed that nucleic acid damage from oxidative stress was highest in patients with dementia, followed by patients with psychotic disorders [8]. Therefore, antioxidant compounds are of interest as potential novel therapeutics to reduce oxidative stress in psychiatric and neurodegenerative diseases.

Plant-derived compounds such as phenolic, flavonic, and anthocyanic derivatives are increasingly investigated for their potential to benefit multiple aspects of health through anti-inflammatory and antioxidant effects [9,10,11]. Phenols represent a large group of compounds that are structurally characterised by at least one aromatic ring with one or more hydroxyl groups attached [12] and are abundant in plants, including fruits and vegetables [13]. Phenolic compounds act as antioxidants by reacting with free radicals through hydrogen atom transfer, the transfer of a single electron, sequential proton loss electron transfer, and the chelation of pro-oxidant metal ions [14]. 

Flavonoids are the most prevalent class of dietary phenolic compounds [15,16]. Flavonoids can be divided into two major categories based on the saturation of the central heterocyclic ring; i.e., flavonols, flavones, and anthocyanidins are unsaturated flavonoids, whereas flavan-3-ols, flavanones, and dihydroflavonols are examples of saturated flavonoids [17]. Epidemiological evidence has shown positive associations between the dietary intake of flavonoids and cognition among healthy older adults [18] and lower odds of cognitive decline [19]. Preclinical studies have also shown support for the antioxidant properties of flavonoids. For example, kaempferol (100 mg/kg) increased antioxidant pathways in streptozotocin (STZ)-induced diabetic rats [20], and the flavonoid-rich fraction of *Mondodora tenuifola* exerted antidepressant-like effects in a stress-induced rat model of depression where behavioural improvements were accompanied by improved antioxidant enzyme activities such as superoxide dismutase, reduced glutathione and catalase [21]. The interaction between flavonoids and neuronal signalling cascades, such as p-PI3K/Akt/GSK3β and mitogen-activated protein kinase (MAPK) pathways, is a potential mechanism by which flavonoids can regulate pro-survival transcription factors and gene expression, leading to the inhibition of apoptosis and the promotion of neuronal survival and differentiation [22]. Flavonoids also increase peripheral and cerebral vascular blood flow [23,24], which can promote angiogenesis and nerve cell growth and is important for memory and cognition [22]. 

Anthocyanins are a subclass of flavonoids that provide red, purple, and blue pigmentation in plant-based foods [25], such as red cabbage, blueberries, blackcurrants, mulberries, and cherries [26]. Anthocyanins are promising for the treatment of neurological disorders due to their antioxidant and anti-inflammatory effects [25,27,28]. In clinical trials, anthocyanins reduced inflammation in older adults with MCI [9] and improved vascular function and cognition in healthy older individuals [29]. In an amyloid precursor protein/presenilin-1 (APP/PS1) mouse model of Alzheimer’s disease, dietary anthocyanin supplementation (12 mg/kg body weight/day) for 30 days improved performance in the Morris water maze and Y-maze task, as well as memory-related pre- and post-synaptic protein markers [30]. The authors found that anthocyanins regulated the PI3K/Akt/GSK3β pathway and activated the endogenous antioxidant Nrf2/HO-1 pathway and its target genes, reducing the AβO-induced elevation of ROS-mediated oxidative stress and preventing neurodegeneration [30]. Previous studies (in vitro) have shown that phenolic extracts of boysenberries, blackcurrants [31], and anthocyanins from red and black rice [32] can prevent hydrogen peroxide (H_2_O_2_)-induced oxidative damage in SH-SY5Y cells; a human neuroblastoma cell line.

Evidence to date suggests that commonly consumed plant-based products that contain high phenolic levels may be effective at reducing oxidative damage and inflammation in the brain. For example, a previous study from our laboratory demonstrated the efficacy of the Queen Garnet plum (QGP: *Prunus salicina*, known for its particularly high anthocyanin content [33] and antioxidant properties [34,35]) in reducing TNF-α in older adults with MCI [9]. In addition, black pepper (*Piper nigrum*) has antioxidant, anticancer, and antimicrobial properties [36]. Preclinical studies have shown anxiolytic and antidepressant-like effects and improved cognition in rodents [37,38,39,40,41] via reduced hippocampal oxidative stress and neurodegeneration following black pepper administration [37,39]. There is also evidence that sage (*Salvia officinalis*) can improve cognition [42,43] and mood [44] following an acute dose in healthy humans and exert effects similar to diazepam on depression and anxiety in rodents [45,46]. The beneficial effect of elderberry (*Sambucus nigra*) was comparable to diazepam in improving the biochemical and behavioural effects of chronic stress in rodents [47] and showed neuroprotective effects against oxidative and inflammatory responses to cerebral ischemia/reperfusion (I/R) [48]; and improved motor function, inflammation, and oxidative stress in a rodent model of Huntington’s disease [49]. In rodent studies, lemon balm (*Melissa officinalis*) administration produced anxiolytic and antidepressant-like effects in stress-exposed mice [50] and Wistar rats [51] and ameliorated scopolamine-induced learning deficiencies [52] via antioxidant and anti-inflammatory effects. In clinical trials, the chronic administration of lemon balm reduced depression, anxiety, and stress and improved sleep in patients with chronic stable angina [53] and in female adolescents with premenstrual syndrome [54]. In mice, clove (*Syzygium aromaticum*) reduced anxiety- [55] and depression-like behaviours [56], attenuated scopolamine-induced deficits in memory acquisition and retention, and reduced malondialdehyde levels in brain samples, indicating a decrease in oxidative stress [57]. In humans, a clove extract capsule (250 mg) reduced peripheral markers of oxidative stress and inflammation in social drinkers after binge drinking [58]. Furthermore, STZ + high-fat diet-induced diabetic rats administered flavonoid-rich astragalus (*Astragalus membranaceus*, an adaptogenic plant utilised in traditional Chinese Medicine) displayed increased blood–brain barrier integrity, the brain-derived neurotrophic factor (BDNF) and hippocampal mitochondrial biogenesis [59]. 

Overall, the evidence suggests that oxidative stress, inflammation, and neurodegeneration underlie several psychiatric and neurological conditions, while certain plant extracts may benefit oxidative status and behaviours related to cognition, anxiety, and depression. High levels of phenolics may underpin the antioxidant and neuroprotective effects of plants with subsequent benefits on brain health; however, further research is required. Therefore, the aim of this study was to (1) investigate the total phenolic, flavonoid, and anthocyanin content, the antioxidant and metal chelating capacities of key whole plant foods and over-the-counter plant-based medicinal products, and (2) to determine their ability to treat and prevent H_2_O_2_-induced oxidative stress using SH-SY5Y neuroblastoma cells (in vitro).

## 2. Materials and Methods

### 2.1. Plant and Medicine Samples

**Figure 1:**
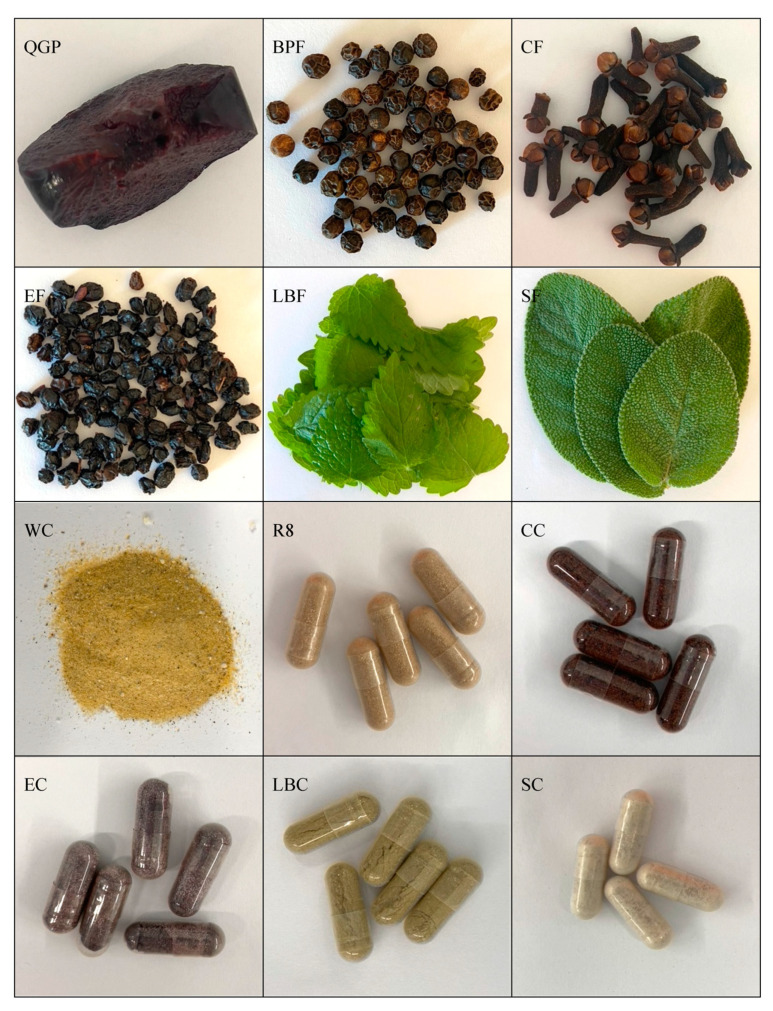
Images of samples prior to extraction. BPF, *P. nigrum*; CC, Fresh Ground Cloves Herbal Supplement, Kroeger Herb^®^ Products Co., Inc.; CF, *S. aromaticum*; EC, Sambucol Black Elderberry Cold and Flu, Pharmacare Laboratories Pty Ltd.; EF, *S. nigra*; LBC, Nature’s Sunshine Lemon Balm, Nature’s Sunshine Products of Australia Pty Ltd.; LBF, *M. officinalis*; QGP, *P. salicina*; R8, Relax–Stress Relief, Regul8 Pty Ltd.; SC, Hilde Hemmes’ Herbals Sage 1000 capsule, Herbal Supplies Pty Ltd.; SF, *S. officinalis;* WC, WelleCo Super Boosters Immune system support with Kakadu Plum, Welle Pty Ltd.

Plant samples were examined for their total phenolic, flavonoid, and monomeric anthocyanin content, oxygen and nitrogen radical scavenging, and copper and iron ion-chelating capacities. The physiological antioxidant potential of samples was then examined in SH-SY5Y cells (in vitro) using prevention and treatment paradigms. Plant samples, including QGP (*P. salicina*); black pepper (*P. nigrum*); clove (*S. aromaticum*); elderberry (*S. nigra*); lemon balm (*M. officinalis*); and sage (*S. officinalis*), are detailed in Table 1. QGP was used as a comparator due to evidence demonstrating high anthocyanin content, and antioxidant and anti-inflammatory capacities [34,35,60], including results from our clinical trial [9]. Over-the-counter plant-based medicine products were also examined; two of these medicinal products contained a blend of plant extracts, while the other samples contained one primary active ingredient (*S. aromaticum*, *S. nigra*, *M. officinalis*, *S. officinalis*) detailed in Table 2. Images of samples prior to extraction are presented in Figure 1.

### 2.2. Extraction Procedure

Plant and medicinal extracts were prepared using previously published methods [61,62]. Briefly, the samples were snap-frozen with liquid nitrogen and ground using a mortar and pestle. Samples were extracted in acidified methanol (MeOH:H_2_O:HCl) for the colourimetric assays and dimethyl sulfoxide (DMSO) for the cell-based assays. Ground samples (300 mg) were combined with a solvent (3 mL), sonicated (15 min), and centrifuged (5 min at 5000 rpm; Sorvall ST 8 Small Benchtop Centrifuge, Thermo Scientific, Melbourne, VIC, Australia, used throughout the present study). The supernatant was then removed, and the extraction process was repeated twice further, resulting in 9 mL of extract in total. The combined supernatants were filtered using a 0.45 μm membrane and stored at −80 °C until further analyses. All efforts were made to ensure minimal light exposure throughout the experiments. All experiments were conducted in triplicate (minimum). 

### 2.3. Determination of Total Phenolic Content (TPC)

The colorimetric Folin–Ciocalteu (F-C) assay was used to determine the TPC of the samples, based on Musci and Yao [63]. Samples and standards were added to a 96-well plate, followed by ultrapure water (milliQ^®^ water, Merk, NJ, USA) (H_2_O) and the F-C reagent (diluted 1:1 with H_2_O). After incubation (5 min), a saturated sodium carbonate (Na_2_CO_3_) solution (9% *w*/*v*) was added. The plate was incubated (1 h) and then absorbance was measured (765 nm) using a spectrophotometer (FLUOstar omega filter-based multi-mode, BMG Labtech, Ortenberg, Germany, used throughout the present study). Raw data were blank corrected and the results were interpolated using a 14-point standard curve generated from gallic acid (0–300 μg/mL). Results were expressed as TPC in mg of gallic acid equivalents per 100 g of fresh weight (mg GAE/100 g FW) and presented as mean and standard error (±SEM).

### 2.4. Determination of Flavonoid Content

Flavonoid content was determined using two colorimetric assay methods, including the aluminium chloride method (procedure 1), which is selective for flavonols and flavones, and the sodium nitrate method (procedure 2), which is selective for flavan-3-ols [64].

#### 2.4.1. Procedure 1

Aluminium chloride (AlCl_3_, 2% *w*/*v*) and 1 M sodium acetate (CH_3_COONa) were added to the standards and samples in a 96-well plate. The plate was incubated (10 min), and absorbance was measured (425 nm). Raw data were blank corrected, and results were interpolated from a 13-point standard curve generated from quercetin standards (0–475 μg/mL). Results were expressed as the mg of quercetin equivalents per 100 g of fresh weight (mg QCTE/100 g FW) and presented as the mean ± SEM. 

#### 2.4.2. Procedure 2

Sodium nitrite solution (NaNO_2_, 5% *w*/*v*) was added to standards and samples and incubated (5 min). Aluminium chloride (AlCl_3_) was added and the plate was incubated (6 min). A sodium hydroxide (1 M NaOH) solution was then added and after incubation (10 min), the absorbance was measured (510 nm). Raw data were blank corrected, and results were interpolated using a 10-point standard curve generated from catechin standards (0–350 μg/mL). Results were expressed as mg catechin equivalents per 100 g of fresh weight (mg CAE/100 g FW) and presented as the mean ± SEM.

### 2.5. Determination of Monomeric Anthocyanin Content (MAC)

MAC was determined by the pH differential method [65]. Samples and the cyanidin-3-glucoside chloride (C3G) standard were incubated with either a 0.025 M potassium chloride (KCl; pH 1.0) buffer (Buffer A) or 0.4 M sodium acetate (CH_3_CO_2_Na·3H_2_O; pH 4.5) buffer (Buffer B). After incubation (20 min), absorbance was read (520 and 700 nm). Raw data were blank corrected, and the following calculation was applied to quantify the MAC of the samples:

MAC = (A × MW × DF × 10^3^)/(ε × λ)

where A = (520–700 Buffer A) − (520–700 Buffer B), MW = 449.2 g/mol cyanidin-3-glucoside, DF = dilution factor, λ = pathlength in cm, ε = 26,900 molar extraction coefficient for cyanidin-3-glucoside L/mol/cm). Results were expressed as the mg of cyanidin-3-glucoside equivalents per 100 g of fresh weight (mg C3GE/100 g FW) and presented as mean ± SEM.

### 2.6. Determination of Oxygen Radical Absorbance Capacity (ORAC)

The ORAC of the samples was determined by measuring the ability of the sample to delay the degradation of the target molecule, pyrogallol red (PGR), by an oxygen-free radical source (2,2′-azobis (2-amidinopropane) dihydrochloride; AAPH) [66]. A phosphate buffer (75 mM, PB) and PGR (64 μM) were added to samples and standards (trolox standards (0–500 μM)) before being incubated (30 min, 37 °C). AAPH (120 mM, 37 °C) was then added to each well. The absorbance was measured (540 nm) every 3 min for 90 min. ORAC was calculated using the following formula:

$$\mathrm{ORAC}=\frac{\left[\mathrm{AUC}-{\;\mathrm{AUC}}^{0}\right]}{\left[{\mathrm{AUC}}_{\mathrm{Trolox}}-{\mathrm{AUC}}^{0}\right]}\;f[\mathrm{Trolox}]$$

where AUC is the area under the curve of the standard/sample, AUC^0^ is the area under the curve of the control, AUC_Trolox_ is the area under the curve in the presence of Trolox, f is the dilution factor, and Trolox is the concentration in μM. Results were expressed as μmol trolox equivalents per 1 g of fresh weight (μmol TE/g FW) and presented as the mean ± SEM.

### 2.7. Determination of DPPH-Free Radical Scavenging Capacity

The ability of the sample to reduce a nitrogen-free radical (1,1-Diphenyl-2-picrylhydrazyl (DPPH)) was determined by adding trolox standards and samples both in 11 concentrations ranging from 0 to 500 μg/mL to a 96-well plate and adding DPPH (1:1). Absorbance was measured (540 nm) every 15 min for 90 min. The radical scavenging activity (inhibition) was then calculated using the formula:

$$\mathrm{Inhibition}\;\mathrm{ratio}\;\left(\%\right)=\left[1-\left(\frac{{\mathrm{Abs}}_{\mathrm{sample}}}{{\mathrm{Abs}}_{\mathrm{control}}}\right)\right]\times 100$$

where Abs_sample_ is the absorbance of the reaction in the presence of a sample or standard (sample/standard + DPPH solution), and Abs_control_ is the absorbance of the control reaction (solvent + DPPH solution). Inhibition ratios (y) were plotted against sample concentrations (x), and AUC was calculated. Non-linear regression was conducted to determine the mean IC50 ± SEM for each sample [67,68]. The trolox equivalent antioxidant capacity (TEAC) [69] could then be calculated using the formula:

$$\mathrm{TEAC}=\frac{\mathrm{IC}50\;\mathrm{of}\;\mathrm{Trolox}\;(\mu g/L)}{\mathrm{IC}50\;\mathrm{of}\;\mathrm{sample}\;\left(\mu g/L\right)}$$

Results were expressed as μg TEAC per 100 g of fresh weight (μg TEAC/100 g FW) and presented as the mean ± SEM.

### 2.8. Determination of Cu^2+^ Chelating Capacity

The ability of samples to chelate copper ions (Cu^2+^) was determined using the pyrocatechol violet (PV) colorimetric method [70,71,72]. Six ethylenediaminetetracetic acid disodium (EDTA-Na_2_) standards (0–200 μg/mL) and 9 dilutions of each sample (0–16,666.6 μg/mL) were added to a 96-well plate. The sodium acetate buffer (50 mmol/L, C_2_H_3_NaO_2_) (pH 6) and 100 mg/L copper sulphate pentahydrate solution (CuSO_4_·5H_2_O) solution were added and incubated (2 min). The PV solution (2 mmol/L) was then added. A blank containing a sample or standard combined with sodium acetate buffer was used to correct for the unequal colour of the sample. Plates were incubated (20 min), and absorbance was measured (632 nm). The Cu^2+^ complex formation (%) was then calculated using the formula:

$${\mathrm{Cu}}^{2+}\mathrm{chelating}\;\mathrm{ability}\;\left(\%\right)=\left[\frac{\left({\mathrm{Abs}}_{\mathrm{std}0}-{\mathrm{Abs}}_{\mathrm{sample}}\right)}{{\mathrm{Abs}}_{\mathrm{std}0}}\right]\times 100$$

where Abs_std0_ is the absorbance of the reaction in the absence of EDTA-Na_2_ (standard 0), and Abs_sample_ is the absorbance of the reaction in the presence of a sample or standard. Cu^2+^ chelating ability (y) was plotted against sample concentrations (x), and AUC was calculated. Non-linear regression was conducted to determine the mean IC50 ± SEM for each sample (the concentration of the sample that could chelate 50% of Cu^2+^ ions). The EDTA-Na_2_ equivalent (EDTAE) values could then be calculated via the formula:

$$\mathrm{EDTAE}=\frac{\mathrm{IC}50\;\mathrm{of}\;\mathrm{EDTA}\;(\mu g/\mathrm{mL})}{\mathrm{IC}50\;\mathrm{of}\;\mathrm{sample}\;\left(\mu g/\mathrm{mL}\right)}$$

Results were expressed as μg EDTAE per 100 g of fresh weight (μg EDTAE/100 g FW) and presented as the mean ± SEM.

### 2.9. Determination of Fe^2+^ Chelating Capacity

The ability of samples to chelate iron ions (Fe^2+^) was determined using the ferrozine colorimetric method [70,71,72]. Six EDTA-Na_2_ standards (0–50 μg/mL) and 9 dilutions of each sample (0–16,666.6 μg/mL) were added to a 96-well plate, followed by H_2_O and ferrous sulfate (0.3 mmol/L, FeSO_4_) solution. Plates were incubated (5 min), and 0.8 mmol/L ferrozine solution was added, followed by further incubation (10 min). A blank containing a sample or standard combined with H_2_O was used to correct the unequal colour of the sample. Absorbance was measured (562 nm). The formation of the Fe^2+^ complex was calculated using the formula:

$${\mathrm{Fe}}^{2+}\mathrm{chelating}\;\mathrm{ability}\;\left(\%\right)=\left[\frac{\left({\mathrm{Abs}}_{\mathrm{std}0}-{\mathrm{Abs}}_{\mathrm{sample}}\right)}{{\mathrm{Abs}}_{\mathrm{std}0}}\right]\times 100$$

where Abs_std0_ is the absorbance of the reaction with standard 0 (0 mg/L EDTA-Na_2_), and Abs_sample_ is the absorbance of the reaction in the presence of a sample or standard. Fe^2+^ chelating ability (y) was plotted against sample concentrations (x), and AUC was calculated. Non-linear regression was conducted to determine the mean IC50 ± SEM for each sample (the concentration of the sample that could chelate 50% of Fe^2+^ ions). EDTA-Na_2_ equivalent (EDTAE) values were then calculated via the formula:

$$\mathrm{EDTAE}=\frac{\mathrm{IC}50\;\mathrm{of}\;\mathrm{EDTA}\;(\mu g/\mathrm{mL})}{\mathrm{IC}50\;\mathrm{of}\;\mathrm{sample}\;\left(\mu g/\mathrm{mL}\right)}$$

Results were expressed as μg EDTAE per 100 g of fresh weight (μg EDTAE/100 g FW) and presented as the mean ± SEM.

### 2.10. Analysis of In Vitro Effects of Samples on H_2_O_2_-Induced Oxidative Stress Using SH-SY5Y Cells

#### 2.10.1. Cell Culture and Growth

Human neuroblastoma SH-SY5Y (ATCC CRL-2266) cells were cultured using a procedure based on Wang et al. [73]. SH-SY5Y cells were cultured in Dulbecco’s modified eagle medium (DMEM)/F12 supplemented with 10% heat-inactivated fetal bovine serum (FBS) and 1% penicillin/streptomycin (PS) (growth media) from Bovogen Biologicals (Melbourne, VIC Australia). Cells were maintained at 37 °C in a humidified incubator with 5% CO_2_ and 95% relative humidity and passaged every 3 days.

#### 2.10.2. Dose Response of Samples on Cell Viability

The SH-SY5Y cells were seeded in 96-well plates in growth media using a seeding density of 2 × 10^4^ and were incubated (24 h). SH-SY5Y cells were treated with growth media containing different concentrations of sample extracts (10, 25, 50, 100 μg/mL) and were incubated (24 h). Control cells were treated with the growth medium only (no sample). MTT reduction assays, as described below, were used to evaluate the effects of the different concentrations of samples on the viability of the SH-SY5Y cells.

#### 2.10.3. Dose Response of Oxidative Stressor on Cell Viability

The SH-SY5Y cells were seeded in 96-well plates in the growth media using a seeding density of 2 × 10^4^ (200 µL/well) and were incubated (24 h). For oxidative stress induction, cells were exposed to H_2_O_2_ at varying concentrations (0.1, 0.2, 0.5, and 1 mM) for 24 h. Control cells were treated with the growth medium only (no H_2_O_2_). MTT reduction assays, described below, were used to investigate the effects of the different concentrations of H_2_O_2_ on the viability of SH-SY5Y cells. The appropriate concentration of H_2_O_2_ was determined (with cell survival rate of 50%).

Two experimental paradigms were then employed in the present study to examine the ability of samples to either prevent or treat oxidative damage (in vitro): (1) a prevention assay, where cells were administered to sample extracts prior to oxidative stress (H_2_O_2_) exposure; (2) a treatment assay where cells were exposed to oxidative stress followed by sample extracts.

#### 2.10.4. Oxidative Stress Prevention Paradigm and Cell Viability MTT Assay

SH-SY5Y cells were pre-treated with growth media containing different concentrations of the sample extracts (10, 25, 50, 100 μg/mL) and were incubated (2 h). After incubation, media were replaced with media containing sample extracts (10, 25, 50, 100 μg/mL) in the presence of H_2_O_2_ (0.15 mM) and were incubated for a further 24 h. MTT (0.5 mg/mL) was then added to each well and incubated (3 h, 37 °C). The media were aspirated, and DMSO (100 µL) was added into each well to dissolve the formazan crystals. Plates were then shaken (15 min), and absorbance was read (570 nm) using a spectrophotometer. Cell viability was expressed as a % of the control (i.e., the absence of a stressor or treatment) and presented as the mean ± SEM.

#### 2.10.5. Oxidative Stress Treatment Paradigm and Cell Viability MTT Assay

SH-SY5Y cells were pre-treated with growth media containing H_2_O_2_ (0.15 mM) and incubated (2 h). After incubation, media were replaced with media containing sample extracts (10, 25, 50, 100 μg/mL) in the presence of H_2_O_2_ (0.15 mM) and were incubated for a further 24 h. MTT (0.5 mg/mL) was then added to each well and incubated (3 hrs, 37 °C). Media were then aspirated, and DMSO (100 µL) was added to dissolve the formazan crystals. Plates were then shaken (15 min), and absorbance was read (570 nm) using a spectrophotometer. Cell viability was expressed as a % of the control (i.e., absence of stressor or treatment) and presented as the mean ± SEM.

### 2.11. Statistical Analysis

All statistical analyses were conducted using IBM SPSS Statistics (Version 29; SPSS Inc., Chicago, IL, USA) and PRISM (version 9 for macOS; GraphPad Software Inc., San Diego, CA, USA). For results that were converted to fresh weight equivalents, the concentration was converted from mg per litre of the extract to mg per 100 g of fresh weight using the formula:

$$\begin{array}{l} \mathrm{Concentration}\;(\mathrm{mg}/100\;g\;\mathrm{FW})\\ \;=(\frac{\mathrm{concentraton}\;(\mathrm{mg}/L)\;\times \;\mathrm{solvent}\;\mathrm{volume}\;(\mathrm{mL})/1000}{\mathrm{fresh}\;\mathrm{weight}\;\mathrm{equivalent}\;\mathrm{of}\;\mathrm{sample}\;\mathrm{weight}\;(g)})\times 100 \end{array}$$

The results expressed by the sample weight can be found in the Supplementary Material (Figure S1). For the colourimetric assay results, one-way analysis of variance (ANOVA) and Dunnett t (2-sided) pairwise comparisons were performed to compare samples to a positive control (QGP, which is known to have high dietary phenolics and antioxidant capacity). For the TEAC and EDTAE results, ANOVA with post hoc Tukey’s honest significant difference (HSD) tests were used to determine differences between samples that were able to inhibit ≥ 50%. Correlations were conducted between the mean total phenolic, flavonol and flavone, flavan-3-ol, and anthocyanin content with mean values for ORAC, DPPH, Cu^2+^, and Fe^2+^’s chelating capacity were examined using Spearman correlations. For cell viability experiments, one-way ANOVAs with post hoc Tukey’s HSD tests were used to determine significant differences between cells treated with samples (various concentrations) and controls, including H_2_O_2_ only, and control (untreated) cells. Data were reported as the mean ± SEM (minimum triplicate determinations). Statistical significance was set to *p* < 0.05.

## 3. Results

### 3.1. Total Phenolic, Flavonoid and Monomeric Anthocyanin Content

#### 3.1.1. Total Phenolic Content

**Figure 2:**
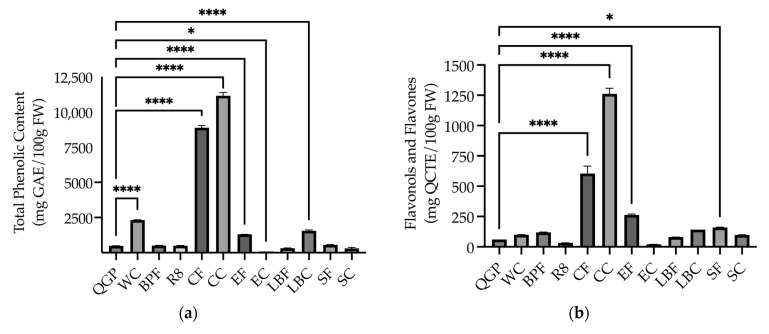

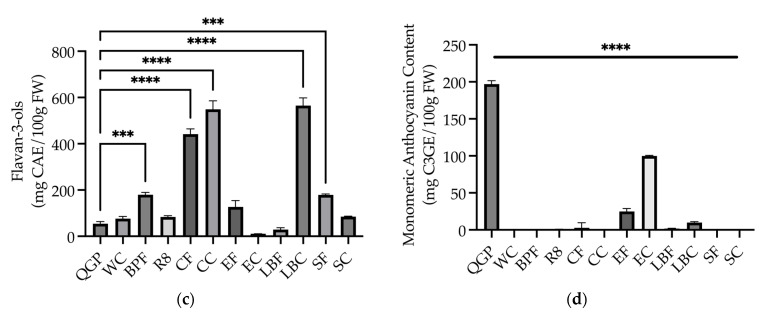
(**a**) Total phenolic (mg gallic acid equivalent (GAE)/100 g fresh weight (FW)); (**b**) flavonol and flavone (mg quercetin equivalent (QCTE)/100 g FW); (**c**) flavan-3-ol (mg catechin equivalent (CAE)/100 g FW); and (**d**) monomeric anthocyanin content (mg cyanidin-3-glucoside equivalent (C3GE)/100 g FW). BPF, *P. nigrum*; CC, Fresh Ground Cloves Herbal Supplement, Kroeger Herb^®^ Products Co., Inc.; CF, *S. aromaticum*; EC, Sambucol Black Elderberry Cold and Flu, Pharmacare Laboratories Pty Ltd.; EF, *S. nigra*; LBC, Nature’s Sunshine Lemon Balm, Nature’s Sunshine Products of Australia Pty Ltd.; LBF, *M. officinalis*; QGP, *P. salicina*; R8, Relax–Stress Relief, Regul8 Pty Ltd.; SC, Hilde Hemmes’ Herbals Sage 1000 capsule, Herbal Supplies Pty Ltd.; SF, *S. officinalis*; WC, WelleCo Super Boosters Immune system support with Kakadu Plum, Welle Pty Ltd. * *p* < 0.05 vs. QGP, *** *p* < 0.001, **** *p* < 0.0001. Data are presented as the mean ± SEM (experiments conducted in triplicate).

A one-way ANOVA revealed a significant difference between the samples (*F*_(11, 24)_ = 2029.61, *p* < 0.001). Dunnett t post hoc analyses showed that WC, CF, CC, EF, and LBC had significantly higher phenolic content than QGP (all *p* < 0.001). BPF, R8, LBF, SF, and SC did not significantly differ in their amount of total phenolics compared to QGP (all *p* > 0.05), while EC had significantly fewer total phenolics than QGP (*p* = 0.016) (Figure 2a).

#### 3.1.2. Flavonoids

A one-way ANOVA revealed that there were significant differences in flavonols and flavones across the samples (*F*_(11, 24)_ = 256.69, *p* < 0.001), with significantly higher content in CF, CC, EF (*p* < 0.001) and SF (*p* = 0.034) compared to QGP, and no significant difference between QGP and the remaining extracts (*p* > 0.05) (Figure 2b). There was also a significant difference in flavan-3-ols across the samples (*F*_(11, 24)_ = 119.11, *p* < 0.001) with significantly higher levels in BPF, SF (*p* < 0.001), CF, CC, LBC (*p* < 0.0001) compared to QGP (Figure 2c). There was no significant difference between QGP and the remaining extracts (all *p* > 0.05) (Figure 2c).

#### 3.1.3. Monomeric Anthocyanin Content

A one-way ANOVA revealed that there was a significant difference in MAC among the samples (*F*_(11, 24)_ = 368.031, *p* < 0.001), with Dunnett t post hoc analyses revealing that all samples had significantly lower MAC compared to the QGP (all *p* < 0.0001) (Figure 2d).

### 3.2. Antioxidant Capacity

#### 3.2.1. ORAC

**Figure 3:**
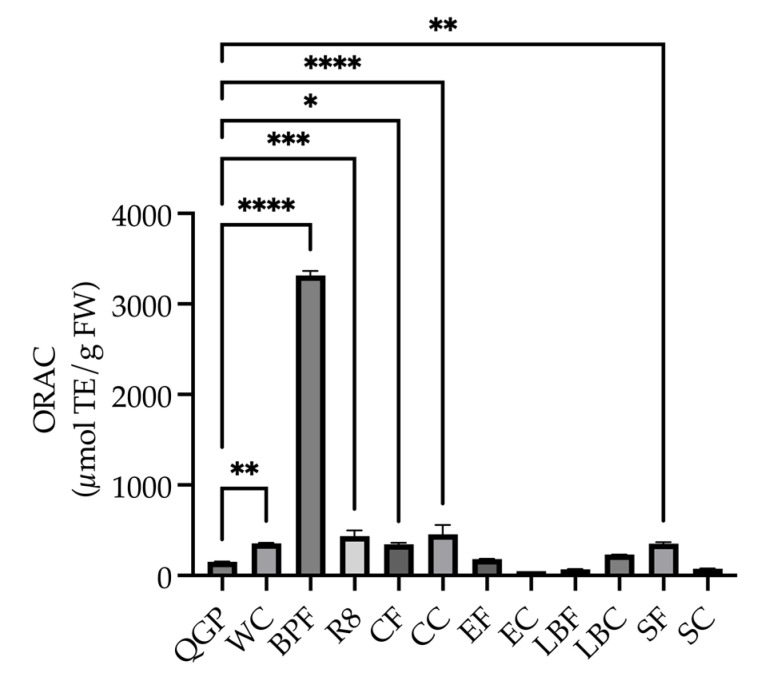
Oxygen radical absorbance capacity (ORAC) of plants and plant-based medicines (μmol trolox equivalent/g fresh weight (FW)). BPF, *P. nigrum*; CC, Fresh Ground Cloves Herbal Supplement, Kroeger Herb^®^ Products Co., Inc.; CF, *S. aromaticum*; EC, Sambucol Black Elderberry Cold and Flu, Pharmacare Laboratories Pty Ltd.; EF, *S. nigra*; LBC, Nature’s Sunshine Lemon Balm, Nature’s Sunshine Products of Australia Pty Ltd.; LBF, *M. officinalis*; QGP, *P. salicina*; R8, Relax–Stress Relief, Regul8 Pty Ltd.; SC, Hilde Hemmes’ Herbals Sage 1000 capsule, Herbal Supplies Pty Ltd.; SF, *S. officinalis;* WC, WelleCo Super Boosters Immune system support with Kakadu Plum, Welle Pty Ltd. * *p* < 0.05 vs. QGP, ** *p* < 0.01, *** *p* < 0.001, **** *p* < 0.0001. Data are presented as the mean ± SEM (experiments conducted in triplicate).

A one-way ANOVA revealed that there was a significant difference in ORAC across the samples (*F*_(11, 24)_ = 555.57, *p* < 0.001) with significantly higher ORAC in WC (*p* = 0.008), BPF (*p* < 0.0001), R8 (*p* < 0.001), CF (*p* = 0.012), CC (*p* < 0.0001) and SF (*p* = 0.009) compared to QGP, and no significant difference between QGP and the remaining extracts (all *p* > 0.05) (Figure 3).

#### 3.2.2. DPPH

**Figure 4:**
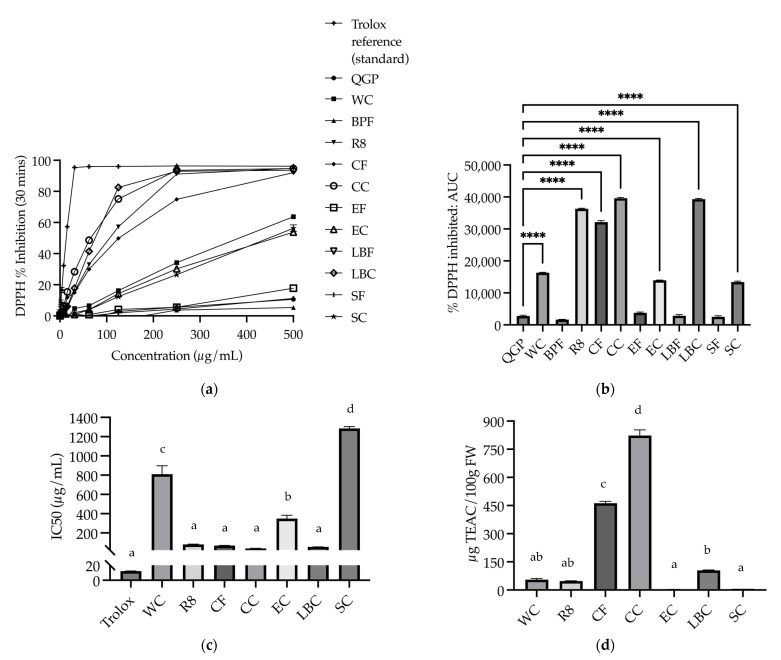
DPPH assay results of plants and plant-based medicines (**a**) % inhibition of oxidation caused by the DPPH free radical of each extract at concentrations in the range of 0–500 μg/mL after 30 min; (**b**) DPPH % inhibition area under the curve (AUC); (**c**) IC50 of extracts that reached 50% inhibition (μg/mL); (**d**) Trolox equivalent antioxidant capacity (TEAC) of extracts (μg TEAC/100 g fresh weight (FW)). BPF, *P. nigrum*; CC, Fresh Ground Cloves Herbal Supplement, Kroeger Herb^®^ Products Co., Inc.; CF, *S. aromaticum*; EC, Sambucol Black Elderberry Cold and Flu, Pharmacare Laboratories Pty Ltd.; EF, *S. nigra*; LBC, Nature’s Sunshine Lemon Balm, Nature’s Sunshine Products of Australia Pty Ltd.; LBF, *M. officinalis*; QGP, *P. salicina*; R8, Relax–Stress Relief, Regul8 Pty Ltd.; SC, Hilde Hemmes’ Herbals Sage 1000 capsule, Herbal Supplies Pty Ltd.; SF, *S. officinalis;* WC, WelleCo Super Boosters Immune system support with Kakadu Plum, Welle Pty Ltd. **** *p* < 0.0001 vs. QGP, or different letters indicate statistically significant differences (minimum *p* < 0.05). Data are presented as the mean ± SEM (experiments conducted in triplicate).

The radical scavenging activity (inhibition percentage) was calculated for extracts of QGP, WC, BPF, R8, CF, CC, EF, EC, LBF, LBC, SF, and SC, and Trolox reference standards. Values of 11 concentrations for each sample, and a standard ranging from 0 to 500 μg/mL were plotted (Figure 4a). The AUC was then calculated for each sample and a one-way ANOVA revealed significant differences between the samples (*F*_(11, 24)_ = 3430.53, *p* < 0.001). Dunnett t post hoc analyses showed that WC, R8, CF, CC, EC, LBC and SC inhibited significantly more oxidation compared to QGP (all *p* < 0.0001) Figure 4b). There was no significant difference between the remaining samples and QGP (*p* > 0.05) (Figure 4b). Non-linear regression was then conducted to determine IC50 values at the endpoint (90 min) for the Trolox reference standards, WC, R8, CF, CC, EC, LBC, and SC extracts, which were the samples able to inhibit ≥ 50% (Figure 4c). IC50 values were used to calculate Trolox equivalent antioxidant capacity (TEAC), and a one-way ANOVA revealed a significant difference in TEAC between the samples (*F*_(6, 12)_ = 558.23, *p* < 0.001) with a significantly higher TEAC in CC than all other samples (*p* < 0.001) (Figure 4d).

### 3.3. Metal Chelating Capacity

#### 3.3.1. Cu^2+^ Chelating

**Figure 5:**
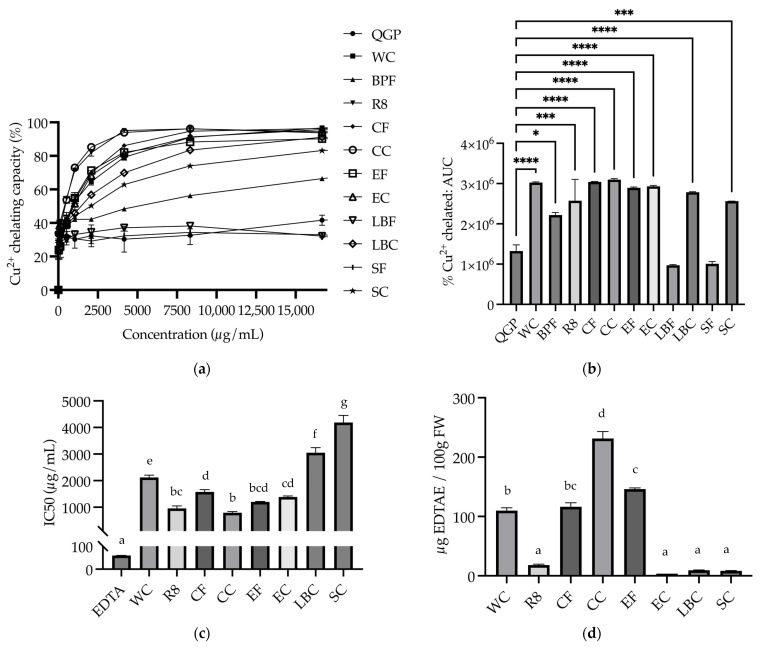
Cu^2+^ chelating capacity of plants and plant-based medicines. (**a**) % of Cu^2+^ chelated by extracts at concentrations in the range of 0–16,666.67 μg/mL; (**b**) % Cu^2+^ chelated area under the curve (AUC); (**c**) IC50 of extracts that reached 50% chelation (μg/mL); (**d**) EDTA-Na_2_ equivalent (EDTAE) Cu^2+^ chelating capacity of extracts (μg EDTAE/100 g fresh weight (FW)). BPF, *P. nigrum*; CC, Fresh Ground Cloves Herbal Supplement, Kroeger Herb^®^ Products Co., Inc.; CF, *S. aromaticum*; EC, Sambucol Black Elderberry Cold and Flu, Pharmacare Laboratories Pty Ltd.; EF, *S. nigra*; LBC, Nature’s Sunshine Lemon Balm, Nature’s Sunshine Products of Australia Pty Ltd.; LBF, *M. officinalis*; QGP, *P. salicina*; R8, Relax–Stress Relief, Regul8 Pty Ltd.; SC, Hilde Hemmes’ Herbals Sage 1000 capsule, Herbal Supplies Pty Ltd.; SF, *S. officinalis;* WC, WelleCo Super Boosters Immune system support with Kakadu Plum, Welle Pty Ltd. * *p* < 0.05 vs. QGP, *** *p* < 0.001, **** *p* < 0.0001, or different letters indicate statistically significant differences (minimum *p* < 0.05). Data are presented as the mean ± SEM (experiments conducted in triplicate).

The percentage of Cu^2+^ chelated by the extracts was calculated for extracts of QGP, WC, BPF, R8, CF, CC, EF, EC, LBF, LBC, SF, and SC and plotted for nine concentrations ranging from 0 to 16,666.67 μg/mL (Figure 5a). The AUC for each sample was calculated, and a one-way ANOVA revealed that there was a significant difference between the samples (*F*_(11, 23)_ = 24.09, *p* < 0.001). WC, CF, CC, EF, EC, LBC (*p* < 0.0001), R8, SC (*p* < 0.001) and BPF (*p* = 0.019) had a significantly larger AUC compared to QGP; however, there was no significant difference between LBF or SF and QGP (*p* > 0.05) (Figure 5b). Non-linear regression was conducted to determine IC50 values for EDTA reference standards and samples. For samples that chelated ≥ 50% of copper ions, IC50 values (Figure 5c) were used to calculate EDTA equivalent values, and one-way ANOVA revealed a significant difference in EDTAE values between the samples (*F*_(8, 17)_ = 157.16, *p* < 0.001). Tukey’s post hoc analyses showed that CC had significantly higher EDTAE than all the other samples (*p* < 0.001) (Figure 5d).

#### 3.3.2. Fe^2+^ Chelating

**Figure 6:**
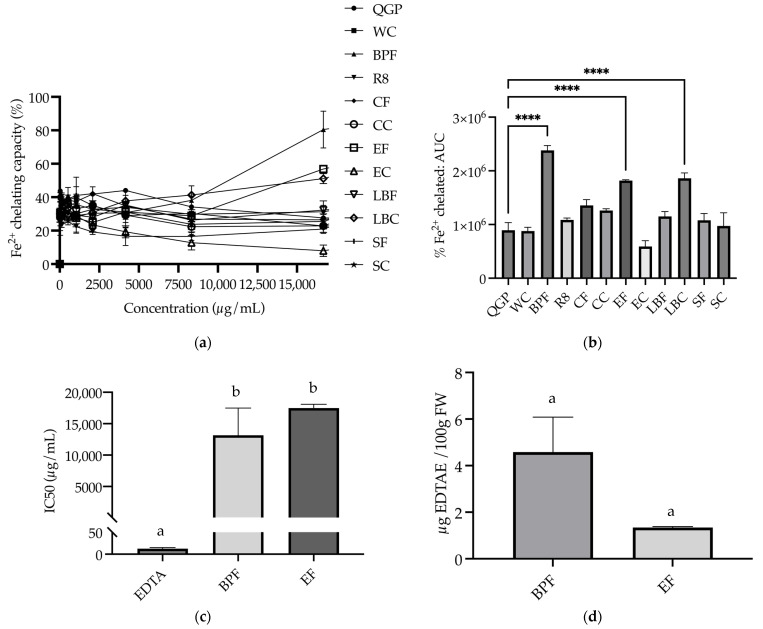
Fe^2+^ chelating capacity of plants and plant-based medicines. (**a**) % Fe2+ chelated by extracts at concentrations in the range of 0–16,666.67 μg/mL; (**b**) % Fe^2+^ chelated area under the curve (AUC); (**c**) IC50 of extracts that reached 50% chelation (μg/mL); (**d**) EDTA-Na_2_ equivalent (EDTAE) Fe^2+^ chelating capacity of extracts (μg EDTAE/100 g fresh weight (FW)). BPF, *P. nigrum*; CC, Fresh Ground Cloves Herbal Supplement, Kroeger Herb^®^ Products Co., Inc.; CF, *S. aromaticum*; EC, Sambucol Black Elderberry Cold and Flu, Pharmacare Laboratories Pty Ltd.; EF, *S. nigra*; LBC, Nature’s Sunshine Lemon Balm, Nature’s Sunshine Products of Australia Pty Ltd.; LBF, *M. officinalis*; QGP, *P. salicina*; R8, Relax–Stress Relief, Regul8 Pty Ltd.; SC, Hilde Hemmes’ Herbals Sage 1000 capsule, Herbal Supplies Pty Ltd.; SF, *S. officinalis*; WC, WelleCo Super Boosters Immune system support with Kakadu Plum, Welle Pty Ltd. **** *p* < 0.0001 vs. QGP, or different letters indicate statistically significant differences (*p* < 0.05). Data are presented as the mean ± SEM (experiments conducted in triplicate).

The percentage of Fe^2+^ chelated by the extracts was calculated for extracts of QGP, WC, BPF, R8, CF, CC, EF, EC, LBF, LBC, SF and SC and plotted for nine concentrations ranging from 0 to 16,666.67 μg/mL (Figure 6a). The AUC for each sample was calculated, and a one-way ANOVA revealed that there was a significant difference between the samples (*F*_(11, 24)_ = 19.95, *p* < 0.001). BPF, EF, and LBC had a significantly larger AUC compared to QGP (*p* < 0.001); however, there was no significant difference between QGP and any of the other extracts (*p* > 0.05) (Figure 6b). Non-linear regression was conducted to determine IC50 values for EDTA reference standards and extracts. For BPF and EF samples that chelated ≥ 50% of iron ions, IC50 values (Figure 6c) were used to calculate the EDTAE values, and a one-way ANOVA revealed no significant difference in EDTAE/100 g FW between the samples (*F*_(1, 3)_ = 8.329, *p* = 0.063) (Figure 6d).

### 3.4. Cell Culture Experiments to Determine Effects of Samples on Oxidative Stress in SH-SY5Y Cells (In Vitro)

#### 3.4.1. Dose Response of Samples on SH-SY5Y Cell Viability in the Absence of Stressor

**Figure 7:**
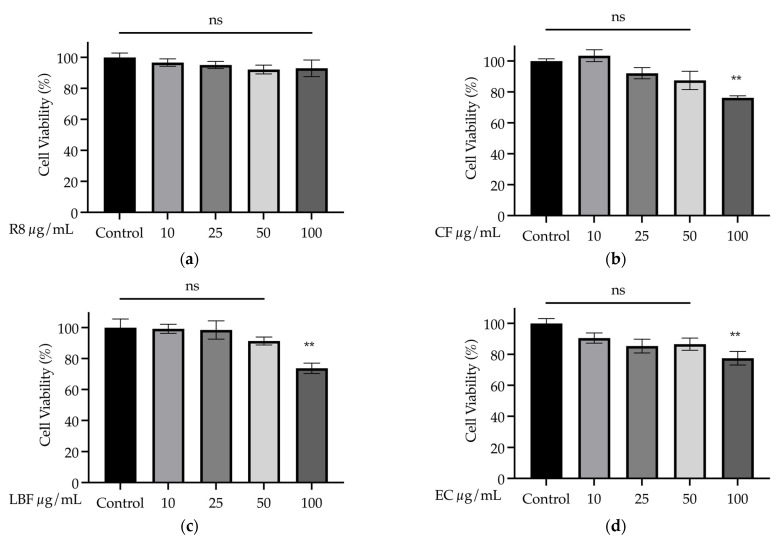

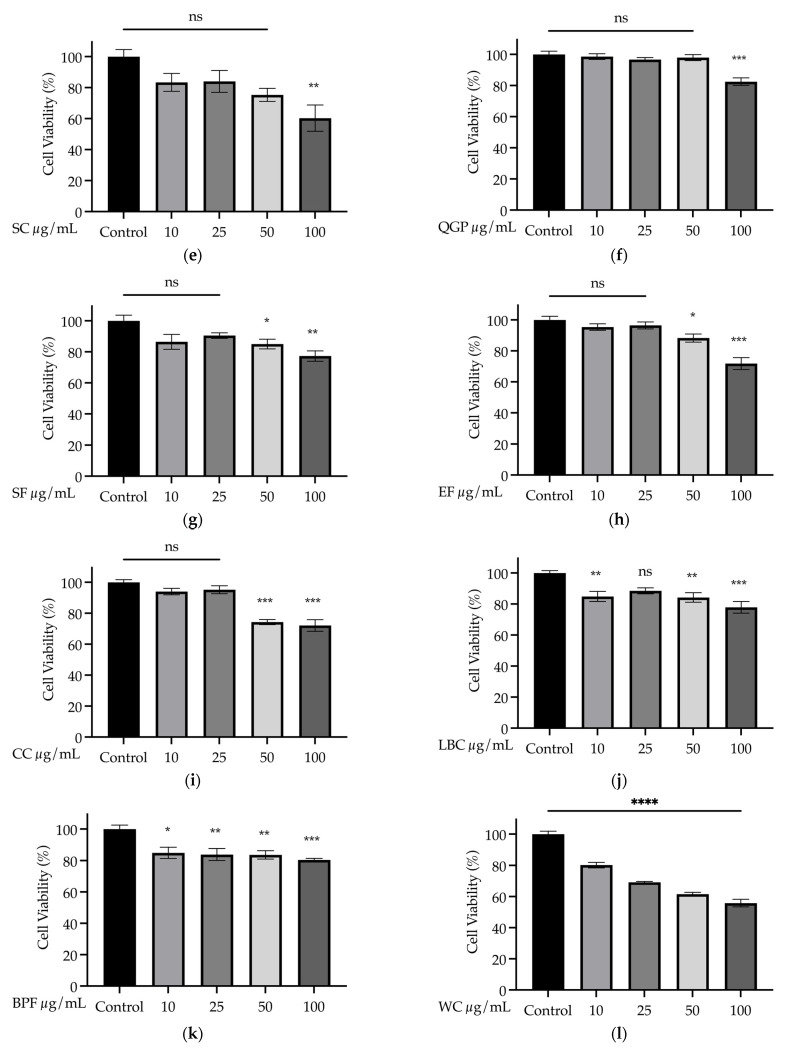
Dose response of plant and plant-based medicine samples (10, 25, 50 and 100 μg/mL) on cell viability (%) in the absence of a stressor in SH-SY5Y cells. (**a**) R8, Relax–Stress Relief, Regul8 Pty Ltd.; (**b**) CF, *S. aromaticum*; (**c**) LBF, *M. officinalis*; (**d**) EC, Sambucol Black Elderberry Cold and Flu, Pharmacare Laboratories Pty Ltd.; (**e**) SC, Hilde Hemmes’ Herbals Sage 1000 capsule, Herbal Supplies Pty Ltd.; (**f**) QGP, *P. salicina*; (**g**) SF, *S. officinalis;* (**h**) EF, *S. nigra*; (**i**) CC, Fresh Ground Cloves Herbal Supplement, Kroeger Herb^®^ Products Co., Inc.; (**j**) LBC, Nature’s Sunshine Lemon Balm, Nature’s Sunshine Products of Australia Pty Ltd.; (**k**) BPF, *P. nigrum*; (**l**) WC, WelleCo Super Boosters Immune system support with Kakadu Plum, Welle Pty Ltd. * *p* < 0.05 vs. untreated healthy controls, ** *p* < 0.01, *** *p* < 0.001, **** *p* < 0.0001, ns = non-significant. Data are presented as the mean ± SEM (*n* = 5 per group).

A one-way ANOVA revealed that the R8 extract did not alter cell viability compared to the non-treated control group (*F*_(4, 20)_ = 0.88, *p* = 0.496) (Figure 7a). On the other hand, five of the examined samples significantly reduced cell viability at the highest dosage (100 μg/mL) (CF (*F*_(4, 20)_ = 8.72, *p* < 0.001), LBF (*F*_(4, 20)_ = 6.69, *p* = 0.001), EC (*F*_(4, 20)_ = 4.48, *p* = 0.010), SC (*F*_(4, 19)_ = 5.39, *p* = 0.004) and QGP (*F*_(4, 20)_ = 13.91, *p* < 0.001)) compared to the non-treated control group (all *p* < 0.01, QGP *p* < 0.001) but no effect on cell viability at lower doses (*p* > 0.05) was observed (Figure 7b–f). There was also the significant effect of SF (*F*_(4, 20)_ = 5.71, *p* = 0.003), EF (*F*_(4, 20)_ = 17.11, *p* < 0.001) and CC (*F*_(4, 20)_ = 27.42, *p* < 0.001) on cell viability, with a significant decrease compared to non-treated controls following the two highest doses (50 and 100 μg/mL) (minimum *p* < 0.05) but no significant difference followed 10 or 25 μg/mL (*p* > 0.05) (Figure 7g–i). LBC significantly decreased cell viability at 10, 50 and 100 μg/mL (*F*_(4, 20)_ = 8.28, *p* < 0.001), while BPF and WC induced significant reductions in cell viability across all doses compared to the untreated controls (*F*_(4, 20)_ = 7.12, *p* < 0.001 and *F*_(4, 20)_ = 104.28, *p* < 0.001, respectively) (Figure 7j,k).

#### 3.4.2. Dose Response of Oxidative Stressor on SH-SY5Y Cell Viability

**Figure 8:**
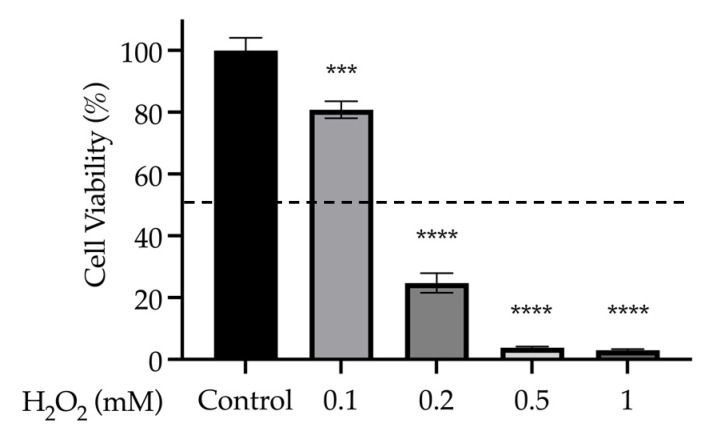
Dose response of H_2_O_2_ oxidative stressor (0.1, 0.2, 0.5 and 1 mM) on cell viability (%) in SH-SY5Y cells. *** *p* < 0.001 vs. untreated healthy controls, **** *p* < 0.0001. Data are presented as the mean ± SEM (*n* = 5 per group). Dotted line represents 50% cell viability.

H_2_O_2_ toxicity in SH-SY5Y cells was determined by exposing cells to concentrations of the oxidant between 0 and 1 mM. A one-way ANOVA showed that all concentrations significantly reduced cell viability compared to the non-treated controls (*F*_(4, 20)_ = 294.43, *p* < 0.001) (all *p* < 0.001) (Figure 8). The concentration of H_2_O_2_ used in subsequent experiments (0.15 mM) was chosen in order to achieve a ~50% reduction in cell viability.

#### 3.4.3. Effect of Samples on Oxidative Stress Prevention in SH-SY5Y Cells (In Vitro)

**Figure 9:**
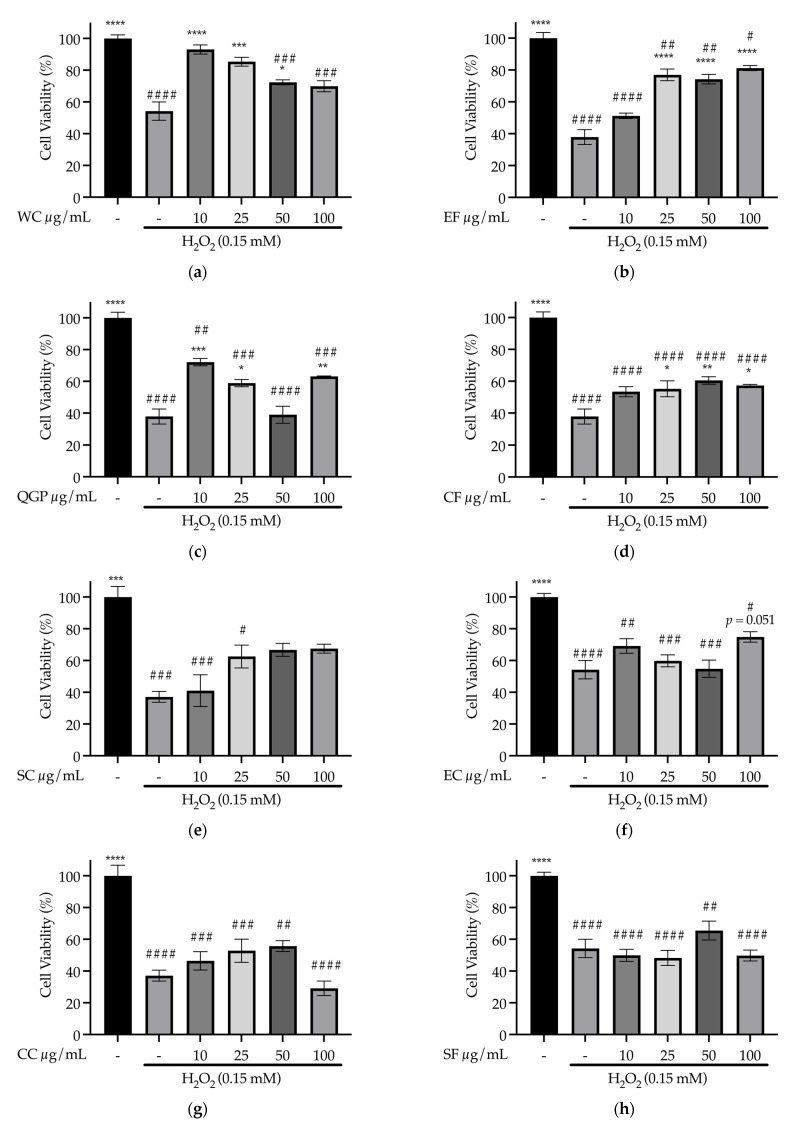

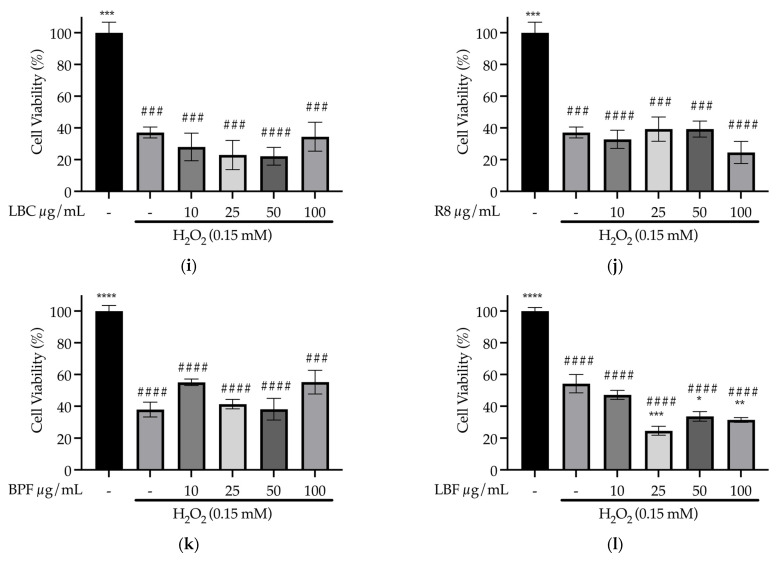
Prevention of H_2_O_2_-induced oxidative stress in SH-SY5Y cells: effect of sample concentration (10, 25, 50 and 100 μg/mL) on cell viability (%) (**a**) WC, WelleCo Super Boosters Immune system support with Kakadu Plum, Welle Pty Ltd.; (**b**) EF, *S. nigra*; (**c**) QGP, *P. salicina*; (**d**) CF, *S. aromaticum*; (**e**) SC, Hilde Hemmes’ Herbals Sage 1000 capsule, Herbal Supplies Pty Ltd.; (**f**) EC, Sambucol Black Elderberry Cold and Flu, Pharmacare Laboratories Pty Ltd.; (**g**) CC, Fresh Ground Cloves Herbal Supplement, Kroeger Herb^®^ Products Co., Inc.; (**h**) SF, *S. officinalis;* (**i**) LBC, Nature’s Sunshine Lemon Balm, Nature’s Sunshine Products of Australia Pty Ltd.; (**j**) R8, Relax–Stress Relief, Regul8 Pty Ltd.; (**k**) BPF, *P. nigrum*; (**l**) LBF, *M. officinalis*. * *p* < 0.05 vs. H_2_O_2_ treatment group, ** *p* < 0.01, *** *p* < 0.001, **** *p* < 0.0001; # *p* < 0.05 vs. untreated control group, ## *p* < 0.01, ### *p* < 0.001, #### *p* < 0.0001. Data are expressed as the mean ± SEM (*n* = 3).

One-way ANOVA revealed a significant effect of WC pre-treatment on cell viability during H_2_O_2_-induced oxidative stress (*F*_(5, 9)_ = 24.64, *p* < 0.001). Compared to the H_2_O_2_ treatment group, there was a significant increase in the viability of cells that received 10 (+38.83%, *p* < 0.001), 25 (*p* < 0.001), and 50 μg/mL (*p* = 0.024) of WC in the extract prior to H_2_O_2_ treatment, but not 100 μg/mL (*p* = 0.059) (Figure 9a). In the 10 and 25 μg/mL WC pre-treatment groups, cell viability did not significantly differ from non-treated healthy controls (*p* = 0.700 and *p* = 0.080, respectively); however, 50 and 100 μg/mL WC groups remained overall lower than the controls (*p* < 0.001) (Figure 9a). Pre-treatment with EF also significantly affected cell viability in the presence of H_2_O_2_-induced oxidative stress (*F*_(5, 12)_ = 47.76, *p* < 0.001), with a significant increase in cell viability following 25, 50, and 100 (+43.28%) μg/mL EF (*p* < 0.001) but not 10 μg/mL (*p* = 0.102) compared to the H_2_O_2_ treatment group; however, EF pre-treatment groups remained lower than the untreated controls (minimum *p* < 0.05) (Figure 9b). The same was found for QGP (*F*_(5, 9)_ = 44.19, *p* < 0.001) and CF (*F*_(5, 12)_ = 34.48, *p* < 0.001); i.e., QGP 10 (+34.12%, *p* < 0.001), 25 (*p* = 0.028), and 100 μg/mL (*p* = 0.009) concentrations significantly improved cell viability compared to the H_2_O_2_ treatment group (but not 50 μg/mL, *p* = 1.0), similar to CF 25 (*p* = 0.042), 50 (+22.59%, *p* = 0.007), and 100 μg/mL (*p* = 0.021) (but not 10 μg/mL, *p* = 0.075); however, all groups remained lower than the untreated controls (minimum *p* < 0.05) (Figure 9c,d). 

SC pre-treatment significantly affected cell viability (*F*_(5, 9)_ = 12.65, *p* < 0.001). Although there was no significant improvement in cell viability following SC pre-treatment (all *p* > 0.05 vs. H_2_O_2_), there was no significant difference between the untreated controls and the 50 and 100 μg/mL SC groups (*p* = 0.070 and *p* = 0.078, respectively) (Figure 9e). On the other hand, pre-treatment with EC, CC, SF, LBC, R8, or BPF did not improve cell viability during H_2_O_2_ administration (all *p* > 0.05 vs. H_2_O_2_), while LBF pre-treatment (25 (*p* < 0.001), 50 (*p* < 0.05) and 100 (*p* < 0.01) μg/mL) significantly impaired cell viability (vs. H_2_O_2_) (Figure 9f–l).

#### 3.4.4. Effect of Samples on Treatment of Oxidative Stress in SH-SY5Y Cells (In Vitro)

**Figure 10:**
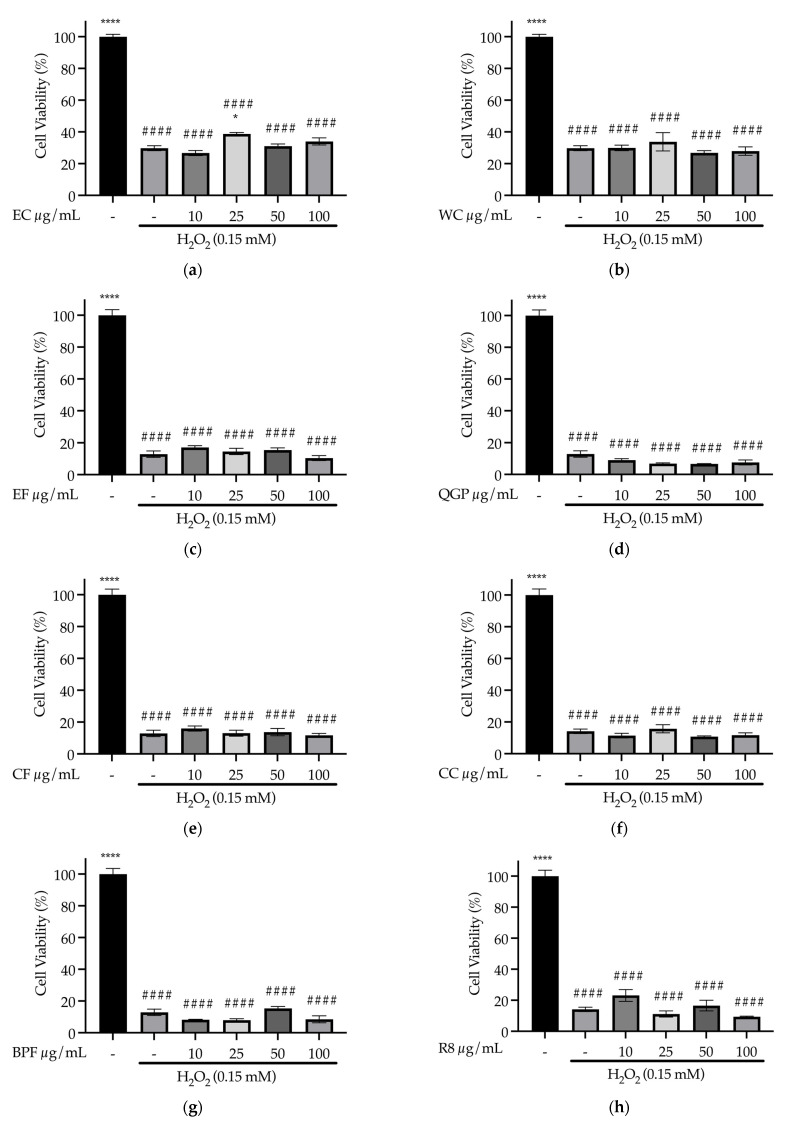

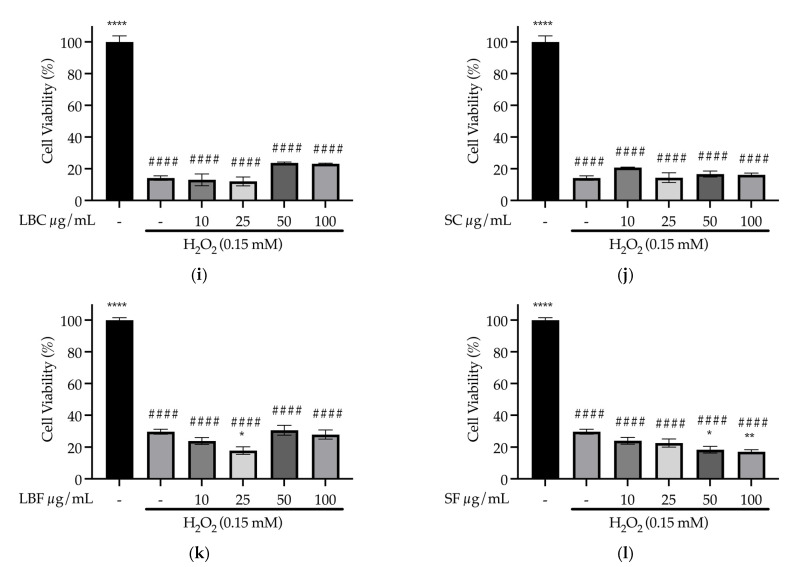
Treatment of H_2_O_2_-induced oxidative stress in SH-SY5Y cells: effect of sample concentration (10, 25, 50 and 100 μg/mL) on cell viability (%) (**a**) EC, Sambucol Black Elderberry Cold and Flu, Pharmacare Laboratories Pty Ltd.; (**b**) WC, WelleCo Super Boosters Immune system support with Kakadu Plum, Welle Pty Ltd.; (**c**) EF, *S. nigra*; (**d**) QGP, *P. salicina*; (**e**) CF, *S. aromaticum*; (**f**) CC, Fresh Ground Cloves Herbal Supplement, Kroeger Herb^®^ Products Co., Inc.; (**g**) BPF, *P. nigrum*; (**h**) R8, Relax–Stress Relief, Regul8 Pty Ltd.; (**i**) LBC, Nature’s Sunshine Lemon Balm, Nature’s Sunshine Products of Australia Pty Ltd.; (**j**) SC, Hilde Hemmes’ Herbals Sage 1000 capsule, Herbal Supplies Pty Ltd.; (**k**) LBF, *M. officinalis*; (**l**) SF, *S. officinalis*. * *p* < 0.05 vs. H_2_O_2_ treatment group, ** *p* < 0.01, **** *p* < 0.0001; #### *p* < 0.0001 vs. untreated control group. Data are presented as the mean ± SEM (*n* = 3).

Treatment with the EC extract had a significant effect on cell viability after H_2_O_2_-induced oxidative stress (*F*_(5, 12)_ = 317.38, *p* < 0.001), with a significant increase in cell viability following 25 μg/mL of EC treatment compared to H_2_O_2_ alone (*p* = 0.016); however, this remained significantly lower than the non-treated controls (*p* < 0.001), demonstrating partial treatment efficacy (Figure 10a). The remaining samples did not improve cell viability compared to the H_2_O_2_ treatment group (statistics are shown in Table S1) (Figure 10b–l).

#### 3.4.5. Correlations

**Figure 11:**
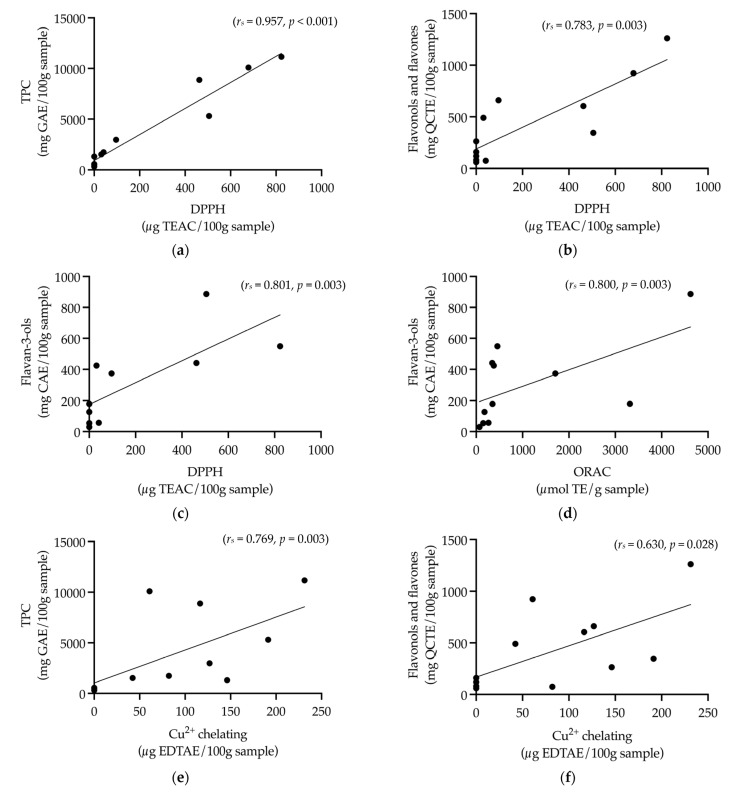
Correlations between the means of samples: BPF, *P. nigrum*; CC, Fresh Ground Cloves Herbal Supplement, Kroeger Herb^®^ Products Co., Inc.; CF, *S. aromaticum*; EC, Sambucol Black Elderberry Cold and Flu, Pharmacare Laboratories Pty Ltd.; EF, *S. nigra*; LBC, Nature’s Sunshine Lemon Balm, Nature’s Sunshine Products of Australia Pty Ltd.; LBF, *M. officinalis*; QGP, *P. salicina*; R8, Relax–Stress Relief, Regul8 Pty Ltd.; SC, Hilde Hemmes’ Herbals Sage 1000 capsule, Herbal Supplies Pty Ltd.; SF, *S. officinalis*; WC, WelleCo Super Boosters Immune system support with Kakadu Plum, Welle Pty Ltd. (**a**) Total phenolic content (TPC) (mg gallic acid equivalent (GAE)/100 g sample) and DPPH (μg trolox equivalent antioxidant capacity (TEAC)/100 g sample), (**b**) flavonols and flavones (mg quercetin equivalent (QCTE)/100 g sample) and DPPH (μg TEAC/100 g sample), (**c**) flavan-3-ols (mg catechin equivalent (CAE)/100 g sample) and DPPH (μg TEAC/100 g sample), (**d**) flavan-3-ols (mg CAE/100 g sample) and ORAC (μmol trolox equivalent (TE)/g sample), (**e**) TPC (mg GAE/100 g sample) and Cu^2+^ chelating (μg EDTA-Na_2_ equivalent (EDTAE)/100 g sample), (**f**) flavonols and flavones (mg QCTE/100 g sample) and Cu^2+^ chelating (μg EDTAE/100 g sample).

Correlations were conducted to determine associations between phenolic content assays and performance in metal chelating and oxidation-inhibiting assays. DPPH oxidative capacity was significantly positively correlated with TPC (*r_s_* = 0.957, *p* < 0.001) (Figure 11a), flavonols and flavones (*r_s_* = 0.783, *p* = 0.003) (Figure 11b), and flavan-3-ols (*r_s_* = 0.801, *p* = 0.003) (Figure 11c). Flavan-3-ols were also significantly and positively correlated with ORAC (*r_s_* = 0.800, *p* = 0.003) (Figure 11d). EDTAE copper ion chelating capacity was significantly positively correlated with TPC (*r_s_* = 0.769, *p* = 0.003) (Figure 11e) and flavonols and flavones (*r_s_* = 0.630, *p* = 0.028) (Figure 11f). 

## 4. Discussion

This study investigated the phenolic content, antioxidant, and metal chelating capacity of plant and plant-based over-the-counter medicine extracts and their ability to both prevent and treat oxidation induced in undifferentiated neuroblast-like [74] SH-SY5Y cells (in vitro). The results of the present study show, for the first time, that WC, EF, QGP, and CF pre-treatment significantly prevent a reduction in cell viability resulting from H_2_O_2_-induced oxidative stress in vitro, while EC significantly improved cell viability when administered as a treatment after H_2_O_2_-induced oxidative stress. These results provide preliminary evidence for the preventative (neuroprotective) effects of WC, EF, QGP, and CF against oxidative stress and the ability of EC to assist in the treatment of oxidative stress in the brain. Levels of phenolics, flavonols, and flavones were highest in the clove extracts, flavan-3-ols were highest in the clove extracts and LBC, and anthocyanin content was highest in QGP, EC, and EF, with negligible anthocyanins detected in the remaining samples. Oxygen-free radical scavenging was highest in BPF and, to a lesser extent, in CC, while nitrogen-free radical scavenging was highest in the clove extracts. The highest Cu^2+^ chelating ability was observed in clove and EF, while BPF and EF were weak Fe^2+^ chelators. Correlations showed that oxygen and nitrogen free-radical scavenging and Cu^2+^ chelating were positively associated with phenolics and flavonoids; however, the results of these experiments did not seamlessly coincide with our in vitro studies. 

In the present study, pre-treatment with EF resulted in the highest percent increase in cell viability compared to cells treated with H_2_O_2_ alone (+43.28%). By contrast, the EC extract did not prevent H_2_O_2_-induced cell death; however, this was the only extract that significantly improved cell viability when used as a treatment for oxidative stress (administered post-H_2_O_2_). QGP was also successful in preventing H_2_O_2_-induced cell death (+34.12% cell viability). Given that these extracts contained the highest levels of anthocyanins, the results suggest that anthocyanins may be important for the ability of elderberry and QGP to protect neuronal cells against oxidative stress. This coincides with the existing literature, as several previous studies also reported that extracts high in anthocyanins from plant sources, such as boysenberry and blackcurrant [31], and red and black rice brans [32], are protective against H_2_O_2_-induced oxidative stress in SH-SY5Y cells. However, non-anthocyanin phenolic compounds can also provide protection against oxidative stress in SH-SY5Y cells [75,76], which seems to have been echoed in the present study. For example, CF was highest in phenolics, flavonoids, and nitrogen (DPPH) free radical scavenging capacities and significantly prevented H_2_O_2_-induced cell death. This is consistent with a previous study that reported that clove essential oil and its major component, eugenol, were protective against 6-hydroxdopamine (OHDA)-induced cell death in SH-SY5Y cells [77]. In addition, Liu et al. [78] also reported that clove had higher total phenolic content, flavonoid content, and DPPH scavenging capacity than the majority of 68 Chinese herbal samples tested, including black pepper. Similarly, we found that WC had higher phenolic, oxygen, and nitrogen free radical scavenging and Cu^2+^ chelating levels than the QGP, and WC pre-treatment resulted in cell viability, which was comparable to the untreated controls even in the presence of H_2_O_2_. Our results coincide with the findings of Xu et al. [79], who examined the antioxidant activity of a blend of Astragalus, the main ingredient in WC, and *Paeonia lactiflora*. They found that this combination was effective at preventing reduced cell viability from H_2_O_2_-induced oxidative stress in an MRC-5 cell model and was also able to increase nitrogen-free radical scavenging [79]. Together, these results suggest the role of phenolics and flavonoids in the neuroprotective effects of the CF and WC extract potentially via the involvement of free radical scavenging and heavy metal chelation.

On the contrary, in the present study, BPF was the most effective at reducing oxygen free radicals in the ORAC assay and contained higher levels of flavonoids than the QGP; however, BPF did not significantly improve cell viability during the H_2_O_2_ prevention or treatment paradigms. This result contrasts with a previous [pre-print] study that reported significantly improved SH-SY5Y cell viability following black pepper extract administration during H_2_O_2_-induced oxidative stress, coupled with the decreased H_2_O_2_-induced release of lactate dehydrogenase, PARP1, and caspase-9, suggesting suppressed neuronal toxicity [80]. In addition, Su et al. [81] reported low ORAC and total phenolic levels in black pepper but high DPPH and Fe^2+^ chelating assays, which differed from the present study. These varying results may be due to the methodological differences between the studies as well as seasonal variations in harvesting conditions (discussed further below). Indeed, Walch et al. [82] examined the antioxidant capacity and polyphenolic composition of several commercially available sage teas and found high variation between tea samples for all the parameters measured. The variation between brands demonstrates that sage variety and chemotype, as well as growing and handling conditions, can greatly impact the phenolic profile and antioxidant capacity of these products. Similar to our BPF findings, we showed that, unlike CF, CC did not exert protective effects against H_2_O_2_ in neuronal cells despite CC demonstrating increased phenolics, flavonoids, nitrogen-free radical scavenging, and Cu^2+^ chelating capacity compared to CF. Therefore, our BPF and CC results suggest that flavonoid and free radical scavenging capacity does not always translate to neuroprotection against oxidative stress in vitro. These seemingly confounding findings may be reconciled by the results of another study, which demonstrated that the structure of flavonoids can dictate their neuroprotective and antioxidant effects [83]. Therefore, differences in flavonoid composition may contribute to physiological effects; however, further research characterizing individual phenolic species in samples is required. It is also possible that other compounds in the plant extracts contribute to antioxidant capacity; for example, black pepper contains high levels of β-caryophyllene, a sesquiterpene with antioxidant properties (reviewed in [84,85]) that may provide a non-phenolic mechanism underlying its antioxidant capacity. 

The ability of plant extracts to exert protective effects on cell viability in the presence of H_2_O_2_-induced oxidative stress did not coincide with an ability to improve cell viability in the absence of a stressor. For example, in the presence of H_2_O_2_, WC was able to completely prevent a reduction in cell viability; however, in the absence of the stressor, the same sample significantly reduced cell viability. In fact, most extracts exerted dose-sensitive detrimental effects on cell viability in the absence of oxidative stress in the present study. R8 was the only sample that did not reduce cell viability at any concentration; however, pre-treatment with R8 did not significantly improve cell viability in the presence of H_2_O_2_-induced oxidative stress. Several studies have found that phenolic extracts reduce cell viability in the absence of a stressor; for example, Dhanalakshmi et al. [75] found that vanillin, a natural phenolic compound, reduced cell viability at concentrations >1 μM and Hamdi et al. [77] found that clove essential oil was toxic at concentrations >40 μg/mL. Together with the existing studies, these results suggest that plant-derived extracts administered directly to cells without a stressor can have differential effects despite being protective in the presence of a physiological stressor. This finding is in line with the idea that medicines can be detrimental if used for reasons other than the intended disease indicated. Further research examining the potential safety implications of consuming plant-based extracts and medicines in the absence of a pathological presentation could be beneficial.

A limitation of this study is the use of the SH-SY5Y cell line. Although this cell line is robust and a commonly published model to examine pathophysiological and pharmacological effects [74], further experiments utilising pluripotent stem cells derived from key patient groups, e.g., individuals with dementia or psychiatric disorders, could bring in vitro results closer to a clinical scenario [86]. In addition, an examination of the treatment and preventative effects of plant-based medicines on microglia, which regulate the immune response in the brain and might be key to oxidative stress [87], would also be important. Another limitation of plant-based research is that numerous factors can influence the concentration of compounds in plants, including nutrition, humidity, temperature, the age of the plant, strain, harvest time, plant stress, manufacturing processes, and storage conditions (e.g., plant bioactive compounds can be heat and light sensitive) [82,88,89,90,91,92]. Therefore, the variability of plant phytochemical composition can limit the generalisability of the results. Furthermore, heterogeneity between the results of studies can also be attributed to variations in methodologies [93]. Establishing standardised protocols to assess antioxidant effects could enhance the comparability of research findings. In addition to exogenous antioxidant effects, further research should also examine the effects of plant-based medicines on the endogenous antioxidant system, as increased antioxidant enzyme activity, protein expression, and the inhibition of caspase-3 activity have been reported [80,94]. 

In conclusion, we report that phenolic-rich plant and plant-based medicines are neuroprotective against oxidative damage in neuronal-like (SH-SY5Y) cells in vitro. Our study demonstrates a link between phenolics, free radical scavenging and metal chelating capacities, and neuroprotective effects during oxidative stress. The results demonstrate the differential effects of the extracts on cell viability in the presence of oxidative stress compared to a healthy state. Further studies examining the safety and efficacy of phytochemical compounds in a clinical setting are justified and may have relevance to addressing oxidative damage in neurological, neuropsychiatric, and neurodegenerative illnesses. 

## Figures and Tables

**Table 1:** Plant sample details.

Sample ID	Name	Scientific Name	Form	Place of Origin
QGP	Queen Garnet Plum	*Prunus salicina*	Fresh	QLD, Australia
BPF	Black Peppercorn	*Piper nigrum*	Dried	India
CF	Clove	*Syzygium aromaticum*	Dried	Madagascar
EF	Elderberry	*Sambucus nigra*	Dried	Germany
LBF	Lemon Balm	*Melissa officinalis*	Fresh	Illawarra Region, NSW, Australia
SF	Sage	*Salvia officinalis*	Fresh	Illawarra Region, NSW, Australia

**Table 2:** Over-the-counter plant-based medicine sample details.

Sample ID	Name of Product	Active Ingredients	Form
WC	WelleCo Super Boosters Immune system support with Kakadu Plum, Welle Pty Ltd., Melbourne, VIC, Australia.	Astragalus (*Astragalus membranaceus* extract 500 mg) equiv. to dry root 1.4 g; Echinacea (*Echinacea purpurea* extract 200 mg) equiv. to dry root 400 mg; Acerola (*Malpighia glabra* extract 442 mg) equiv. to fresh fruit 4.4 g (standardized to contain vitamin C ((ascorbic acid)) 75.1 mg); Moringa (*Moringa oleifera*) leaf powder 300 mg; Olive (*Olea europaea* extract 111.1 mg) equiv to dry leaf 400 mg; Kakadu plum (*Terminalia ferdinandiana*) fruit flesh powder 412.5 mg (standardized to contain vitamin C (ascorbic acid) 41.3 mg); Total vitamin C 104.8 mg; Ginger (*Zingiber officinale* extract 375 mg) equiv. to dry rhizome 1.5 g.	Powder
R8	Relax–Stress Relief, Regul8 Pty Ltd., Crows Nest, NSW, Australia.	Lemon balm (*M. officinalis*) extract dry concentrate 100 mg, derived from dry leaf and flower 1 g; *Rehmannia glutinosa* extract dry concentrate 100 mg, derived from dry root 1 g; passionflower (*Passiflora incarnata*) extract dry concentrate 37.5 mg, derived from dry herb top flowering 750 mg, rose root (*Rhodiola rosea*) extract dry concentrate 100 mg, derived from dry root 800 mg standardized to rosavin 3 mg; *P. nigrum* (BioPerine^®^, Sabinsa^®^, Sydney, NSW, Australia) 244 μg, derived from dry fruit 5 mg; mekabu seaweed (*Undaria pinnatifida*) extract concentrate 25 mg, derived from dry whole plant 300 mg, standardized to fucoidan 12.5 mg	Capsule
CC	Fresh Ground Cloves Herbal Supplement, Kroeger Herb^®^ Products Co., Inc., Longmont, CO, USA.	Cloves (*S. aromaticum*) 450 mg	Capsule
EC	Sambucol Black Elderberry Cold & Flu, Pharmacare Laboratories Pty Ltd., Mona Vale, NSW, Australia.	Black elderberry (*S. nigra*) fruit juice dry equivalent to fresh juice 3.8 g	Capsule
LBC	Nature’s Sunshine Lemon Balm, Nature’s Sunshine Products of Australia Pty Ltd., Baulkham Hills, NSW, Australia.	Herbal extract standardized equiv. to dry lemon balm (*M. officinalis*) leaf 1.95 g equiv. to Rosmarinic acid 33 mg	Capsule
SC	Hilde Hemmes’ Herbals Sage 1000 capsule, Herbal Supplies Pty Ltd., St. Agnes, SA, Australia.	Extract equivalent to dried sage (*S. officinalis*) leaf 1 g	Capsule

## Data Availability

The data presented in this study can be made available on request.

## Supplementary Materials

The following supporting information can be downloaded at: https://www.mdpi.com/article/10.3390/nu15183912/s1, Figure S1: Colourimetric assay results expressed in grams of sample weight (**a**) Total phenolic content (mg gallic acid equivalent (GAE)/100g sample); (**b**) Flavonols and flavones (mg quercetin equivalent (QCTE)/100g sample); (**c**) Flavan-3-ols (mg catechin equivalent (CAE)/100g sample); (**d**) Monomeric anthocyanin content (mg cyanidin-3-glucoside equivalent (C3GE)/100g sample); (**e**) Oxygen radical absorbance capacity (ORAC) (μmol trolox equivalent (TE)/g sample); (**f**) DPPH assay (μg trolox equivalent antioxidant capacity (TEAC)/100g sample); (**g**) Cu^2+^ chelating (μg EDTA-Na_2_ (EDTAE)/100g sample); (**h**) Fe^2+^ chelating (μg EDTAE/100g sample). BPF, *P. nigrum*; CC, Fresh Ground Cloves Herbal Supplement, Kroeger Herb^®^ Products Co., Inc.; CF, *S. aromaticum*; EC, Sambucol Black Elderberry Cold & Flu, Pharmacare Laboratories Pty Ltd; EF, *S. nigra*; LBC, Nature’s Sunshine Lemon Balm, Nature’s Sunshine Products of Australia Pty Ltd; LBF, *M. officinalis*; QGP, *P. salicina*; R8, Relax – Stress Relief, Regul8 Pty Ltd; SC, Hilde Hemmes’ Herbals Sage 1000 capsule, Herbal Supplies Pty Ltd; SF, *S. officinalis;* WC, WelleCo Super Boosters Immune system support with Kakadu Plum, Welle Pty Ltd. * *p* < 0.05 vs QGP, ** *p* < 0.01, *** *p* < 0.001, **** *p* < 0.0001 or different letters indicate statistically significant differences (minimum *p* < 0.05); data presented as mean ± SEM (experiments conducted in triplicate). Table S1: ANOVA and Tukey HSD results for the effect of samples on treatment of oxidative stress in SH-SY5Y cells.
