# Supplementary material for: Investigating the Therapeutic Potential of Plants and Plant-Based Medicines: Relevance to Antioxidant and Neuroprotective Effects
Source: Nutrients. 2023 Sep 8;15(18):3912. doi: 10.3390/nu15183912 (PMC10535096; doi:10.3390/nu15183912)
Supplement: Supplementary file 1 [file nutrients-15-03912-s001.zip › nutrients-2586239-supplementary.pdf]

nutrients

MDPI

Supplementary Materials:

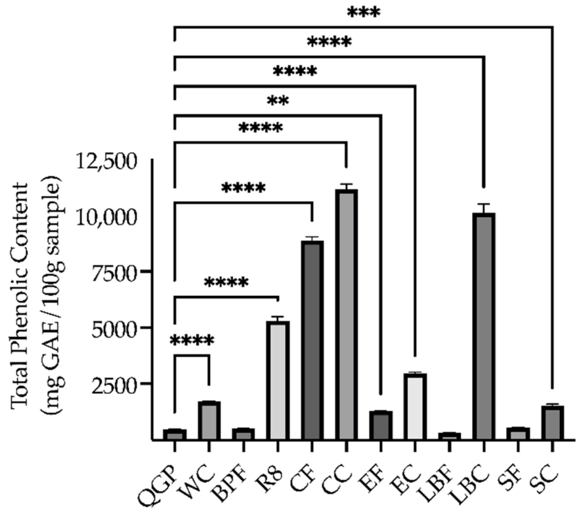

(**a**)

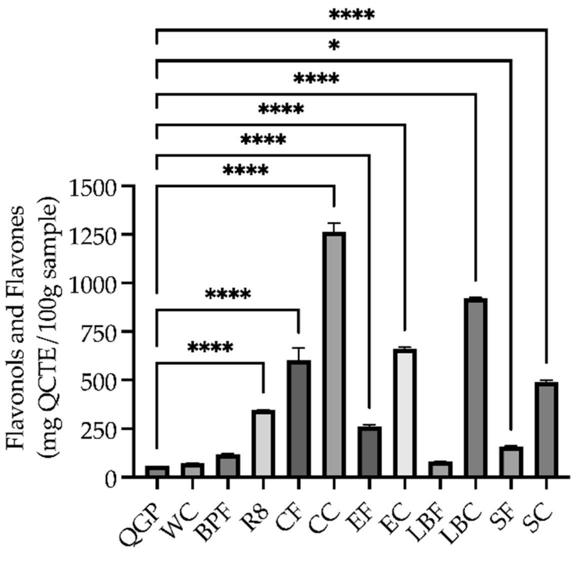

(**b**)

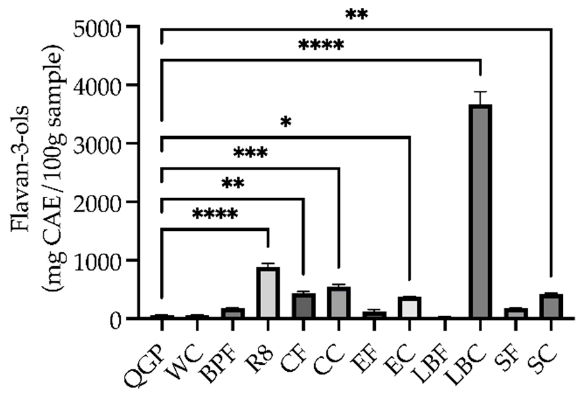

(**c**)

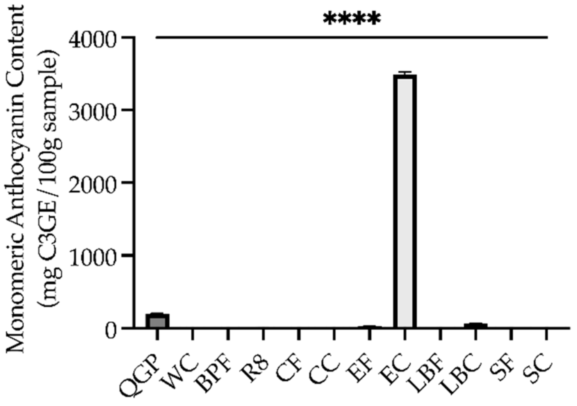

(**d**)

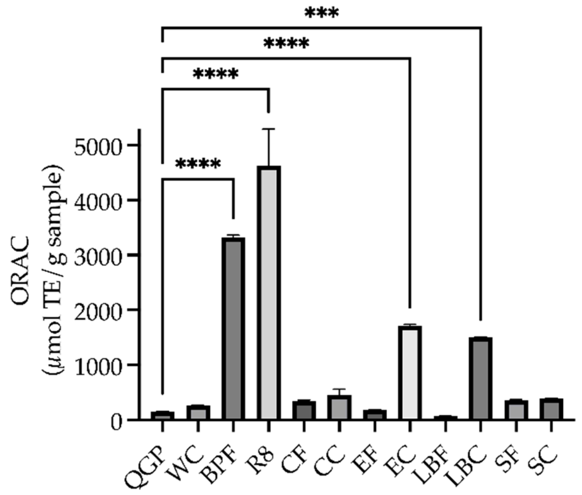

(**e**)

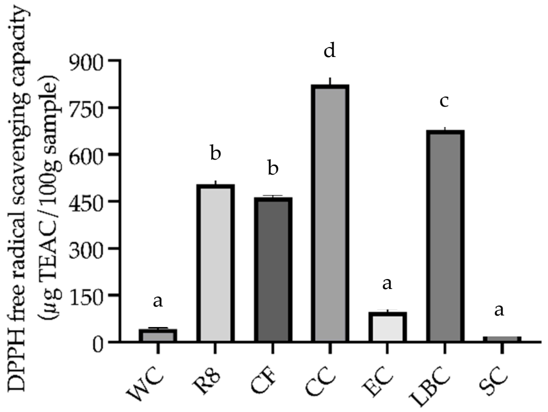

(**f**)

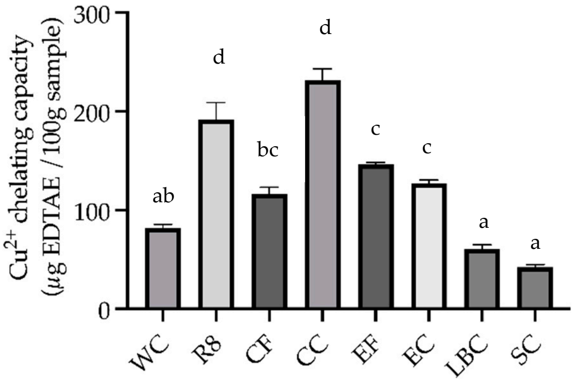

(**g**)

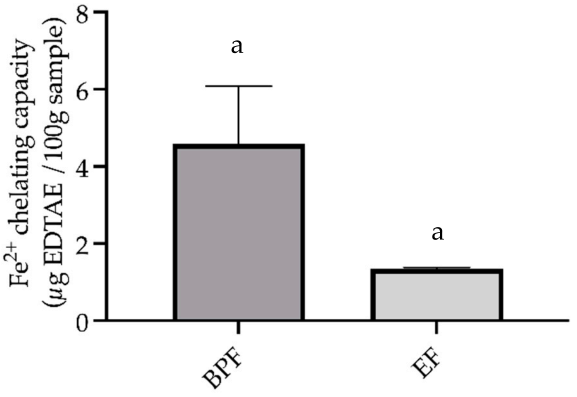

(**h**)

Figure S1: Colourimetric assay results expressed in grams of sample weight (**a**) Total phenolic content (mg gallic acid equivalent (GAE)/100g sample); (**b**) Flavonols and flavones (mg quercetin equivalent (QCTE)/100g sample); (**c**) Flavan-3-ols (mg catechin equivalent (CAE)/100g sample); (**d**) Monomeric anthocyanin content (mg cyanidin-3-glucoside equivalent (C3GE)/100g sample); (**e**) Oxygen radical absorbance capacity (ORAC) (µmol trolox equivalent (TE)/g sample); (**f**) DPPH assay (µg trolox equivalent antioxidant capacity (TEAC)/100g sample); (**g**) Cu^{2+} chelating (µg EDTA-Na_2 (EDTAE)/100g sample); (**h**) Fe^{2+} chelating (µg EDTAE/100g sample). BPF, *P. nigrum*; CC, Fresh Ground Cloves Herbal Supplement, Kroeger Herb® Products Co., Inc.; CF, *S. aromaticum*; EC, Sambucol Black Elderberry Cold & Flu, Pharmacare Laboratories Pty Ltd; EF, *S. nigra*; LBC, Nature's Sunshine Lemon Balm, Nature's Sunshine Products of Australia Pty Ltd; LBF, *M. officinalis*; QGP, *P. salicina*; R8, Relax – Stress Relief, Regul8 Pty Ltd; SC, Hilde Hemmes' Herbals Sage 1000 capsule, Herbal Supplies Pty Ltd; SF, *S. officinalis;* WC, WelleCo Super Boosters Immune system support with Kakadu Plum, Welle Pty Ltd. * $p < 0.05$ vs QGP, ** $p < 0.01$, *** $p < 0.001$, **** $p < 0.0001$ or different letters indicate statistically significant differences ($p < 0.05$); data presented as mean ± SEM (experiments conducted in triplicate).

Table S1. ANOVA and Tukey HSD results for the effect of samples on treatment of oxidative stress in SH-SY5Y cells.

Sample	ANOVA	Tukey's HSD	
		Sample concentration vs H_2O_2	Sample concentration *vs* untreated controls
QGP	$F_{(5, 11)} = 395.30$, $p < 0.001$	All $p > 0.05$	All $p < 0.001$
WC	$F_{(5, 12)} = 99.54$, $p < 0.001$	All $p > 0.05$	All $p < 0.001$
BPF	$F_{(5, 11)} = 357.33$, $p < 0.001$	All $p > 0.05$	All $p < 0.001$
R8	$F_{(5, 12)} = 158.19$, $p < 0.001$	All $p > 0.05$	All $p < 0.001$
CF	$F_{(5, 12)} = 259.97$, $p < 0.001$	All $p > 0.05$	All $p < 0.001$
CC	$F_{(5, 12)} = 277.08$, $p < 0.001$	All $p > 0.05$	All $p < 0.001$
EF	$F_{(5, 12)} = 290.92$, $p < 0.001$	All $p > 0.05$	All $p < 0.001$
EC	$F_{(5, 12)} = 317.38$, $p < 0.001$	25 µg/mL significantly increased ($p = 0.016$), all others $p > 0.05$	All $p < 0.001$
LBF	$F_{(5, 12)} = 169.73$, $p < 0.001$	25µg/mL significantly decreased ($p = 0.034$), all others $p > 0.05$	All $p < 0.001$
LBC	$F_{(5, 12)} = 182.83$, $p < 0.001$	50 ($p = 0.013$) and 100 µg/mL ($p = 0.006$) significantly	All $p < 0.001$

		decreased, 10 and 25 µg/mL<br>$p > 0.05$	
SF	$F_{(5, 12)} = 275.76$, $p < 0.001$	All $p > 0.05$	All $p < 0.001$
SC	$F_{(5, 12)} = 231.15$, $p < 0.001$	All $p > 0.05$	All $p < 0.001$
